# A comprehensive review of machine learning algorithms and their application in geriatric medicine: present and future

**DOI:** 10.1007/s40520-023-02552-2

**Published:** 2023-09-08

**Authors:** Richard J. Woodman, Arduino A. Mangoni

**Affiliations:** 1 Centre of Epidemiology and Biostatistics, College of Medicine and Public Health, Flinders University, GPO Box 2100, Adelaide, SA 5001 Australia; 2 Discipline of Clinical Pharmacology, College of Medicine and Public Health, Flinders University, Adelaide, SA Australia; 3 Department of Clinical Pharmacology, Flinders Medical Centre, Southern Adelaide Local Health Network, Adelaide, SA Australia

**Keywords:** Machine learning, Artificial intelligence, Geriatric medicine, Clinical decisions, Diagnosis, Treatment

## Abstract

The increasing access to health data worldwide is driving a resurgence in machine learning research, including data-hungry deep learning algorithms. More computationally efficient algorithms now offer unique opportunities to enhance diagnosis, risk stratification, and individualised approaches to patient management. Such opportunities are particularly relevant for the management of older patients, a group that is characterised by complex multimorbidity patterns and significant interindividual variability in homeostatic capacity, organ function, and response to treatment. Clinical tools that utilise machine learning algorithms to determine the optimal choice of treatment are slowly gaining the necessary approval from governing bodies and being implemented into healthcare, with significant implications for virtually all medical disciplines during the next phase of digital medicine. Beyond obtaining regulatory approval, a crucial element in implementing these tools is the trust and support of the people that use them. In this context, an increased understanding by clinicians of artificial intelligence and machine learning algorithms provides an appreciation of the possible benefits, risks, and uncertainties, and improves the chances for successful adoption. This review provides a broad taxonomy of machine learning algorithms, followed by a more detailed description of each algorithm class, their purpose and capabilities, and examples of their applications, particularly in geriatric medicine. Additional focus is given on the clinical implications and challenges involved in relying on devices with reduced interpretability and the progress made in counteracting the latter via the development of explainable machine learning.

## Introduction

The recent widespread adoption of electronic health records (EHRs) [1] and broad support from the general public for the sharing of de-identified health records [2], has provided access to an overwhelming volume of health data for research. EHR data include a wide range of information, e.g., images, text, lab data, free text data, claims data, physiological signals and other multimodal information [3]. Multimodal data collected from smart biosensors tracking almost every human physiological system add to the tsunami of Big Health Data [4].

Combined with simultaneous increases in computing power, the explosion in Big Health Data has driven a resurgence in Machine Learning (ML) research including data-hungry Deep Learning algorithms [5–7]. More computationally efficient algorithms with improved performance now offer huge potential for improved diagnosis, risk prediction and more personalised approaches to clinical management [7]. This is particularly relevant for the management of the older patient population which is typically characterised by complex multimorbidity phenotypes and significant interindividual variability in homeostatic capacity, organ function, and response to treatment. Clinical tools that utilise ML algorithms to support clinicians in determining the optimal choice of treatment are slowly gaining the necessary approval from governing bodies and being implemented into healthcare, particularly in the fields of radiology, pathology and imaging, and it is expected that almost all medical disciplines will likely be affected during the next phase of digital medicine [4].

Beyond obtaining regulatory approval, a crucial element in the implementation of these tools into healthcare is the trust and support of the people that use them [8]. An increased understanding by clinicians of artificial intelligence (AI) and ML algorithms provides an appreciation of the benefits, risks, and uncertainties they may bring, and improves the chances for successful adoption [9].

In this article, we first provide a broad taxonomy of ML algorithms, followed by a detailed description of each algorithm class, their purpose and capabilities, and practical applications in healthcare, focusing on geriatric care wherever possible.

## Methods

### Aims of the study

Our main aim is to provide an overview of the types of AI and ML algorithms now available, detail their functioning and purpose and provide examples of their deployment or research within healthcare for older populations. Wherever possible, we provide examples of their use in geriatric care, and where this is not possible, examples of their use for conditions that affect mainly an older population such as cancer or heart failure. Other outcomes included the acceptance of the use of the various technologies by health professionals, and the potential barriers that may prevent their introduction such as ethical and regulatory issues.

### Literature search strategy

We searched the databases of PubMed Central, Semantic Scholar, Google Scholar and arXiv (Cornell University) using suitable terms for both the ML algorithm/architecture of interest and terms for an older and/or multimorbid population. The latter included “older”, “aging”, “aged”, “geriatric care”, “frail”, and “multimorbid”. Publications favoured for inclusion were those that provided a comparison of the algorithm’s performance with a baseline model, provision of accuracy statistics including AUC and/or sensitivity/specificity of higher quality, and more recent publications. We chose to report the AUC where possible since it is a well-established measure of performance in healthcare/medicine and more useful than measures such as overall accuracy (correct classification) which may often be high due to highly imbalanced datasets with a low prevalence of cases. Comparison of the performance with a baseline model enables demonstration of the algorithms ability to be either an improvement upon existing best models or alternatively comparable accuracy but with improvements in other areas such as reduced assessment times and workforce burden.

### Inclusion/exclusion criteria

Given the wide range of machine learning algorithms covered, in places where there was no good example of the application of the algorithm for a much older population, we provide examples in medicine which affect mainly older populations such as cancer, heart failure and dementia. Therefore, we have included studies that had a relevance to an older population and were also able to demonstrate a genuine relevance to either machine learning or AI. We excluded studies that used only a younger population unless we could not find examples of the use of that AI or algorithm in an older population. We included different types of study design including experimental and observational but favoured studies with a larger sample size, especially where this is necessary to provide an adequately sized dataset for the algorithm. We excluded purely qualitative studies. The phenomenon of interest was the novel application of machine learning algorithms within the domain of older medical care which could involve either replacing a previous technique such as a statistical model or be an entirely novel application or AI.

In summary, our paper provides both a broad overview of the topic and sufficient additional knowledge to appreciate the secrets that lie within the various “black boxes”. We also discuss the clinical implications and challenges involved in relying on devices with reduced interpretability and the progress made in counteracting the latter via the development of explainable ML [10].

## Artificial intelligence and machine learning

AI includes the domains of ML, machine reasoning, and robotics. Whilst there is no formal definition of ML it can be considered as the subfield of AI which focuses on the development of algorithms that allow computers to automatically discover patterns in the data and improve with experience, without being given a set of explicit instructions [11]. Rather than being developed or borrowing from one specific scientific field, ML sits at the intersection of statistics, mathematics and computer science, with analytic tools that transcend the boundaries across the three disciplines [5]. A distinct feature of ML algorithms is their data-driven approach to learning, in contrast to rule-based models that rely on domain knowledge. ML algorithms include supervised learning (SL), unsupervised learning (UL), and reinforcement learning (RL) for diagnosis and prediction, phenotyping, and treatment recommendation, respectively [12]. As the name implies, ML algorithms can learn (or improve) by receiving additional data either during training or after deployment. RL algorithms self-learn by using a trial and error approach to determine the best policy including decision making under conditions of uncertainty such as in the context of treatment recommendations [13].

## Model complexity versus clinical interpretability

Earlier approaches to clinical decision support relied on using observational epidemiological data and traditional statistical techniques to develop regression-based risk prediction models such as the Framingham Risk score for the triaging of future cardiovascular events [14, 15]. Although results from these models provide high interpretability by virtue of the model's coefficients, they also have important limitations. Such limitations include assumptions of the underlying data distributions, assumed linear and additive effects, an absence of interactions, a limited number of variables (features), and a dependence on domain expertise. These limitations also lead to a one-size-fits-all population level model designed mainly for identifying the predictive value of risk factors and the average risk for persons with a given combination of those risk factors. As such, the models are unsuitable for individualised risk prediction and more targeted (personalised) approaches to treatment recommendation, key features of the modern management of geriatric patients [16, 17]. Fully incorporating patient heterogeneity including a knowledge of existing disease, physical functioning, and intrinsic capacity requires moving away from previous domain-knowledge modelling approaches to data-driven models that adequately capture the full patient history, demographics, and clinical profile [18, 19]. Clinical decision support tools can, therefore, now be seen to exist on a continuous spectrum of model complexity from regression-based models to state-of-the-art Deep Learning (DL), whose better predictive accuracy is in general traded for their more limited interpretability [5].

## The taxonomy of machine learning

There now exist many thousands of different ML algorithms that have been developed for prediction, pattern recognition or recommendation. The process of model selection, as it is commonly known, involves picking the best algorithm for the specific problem at hand. This is ultimately determined by the broad research goal (e.g., disease diagnosis, risk prediction, phenotyping, or treatment recommendation), the researchers’ knowledge of algorithms that might each fit the purpose, and restrictions arising from the available data such as its volume and dimensionality.

**Fig. 1:**
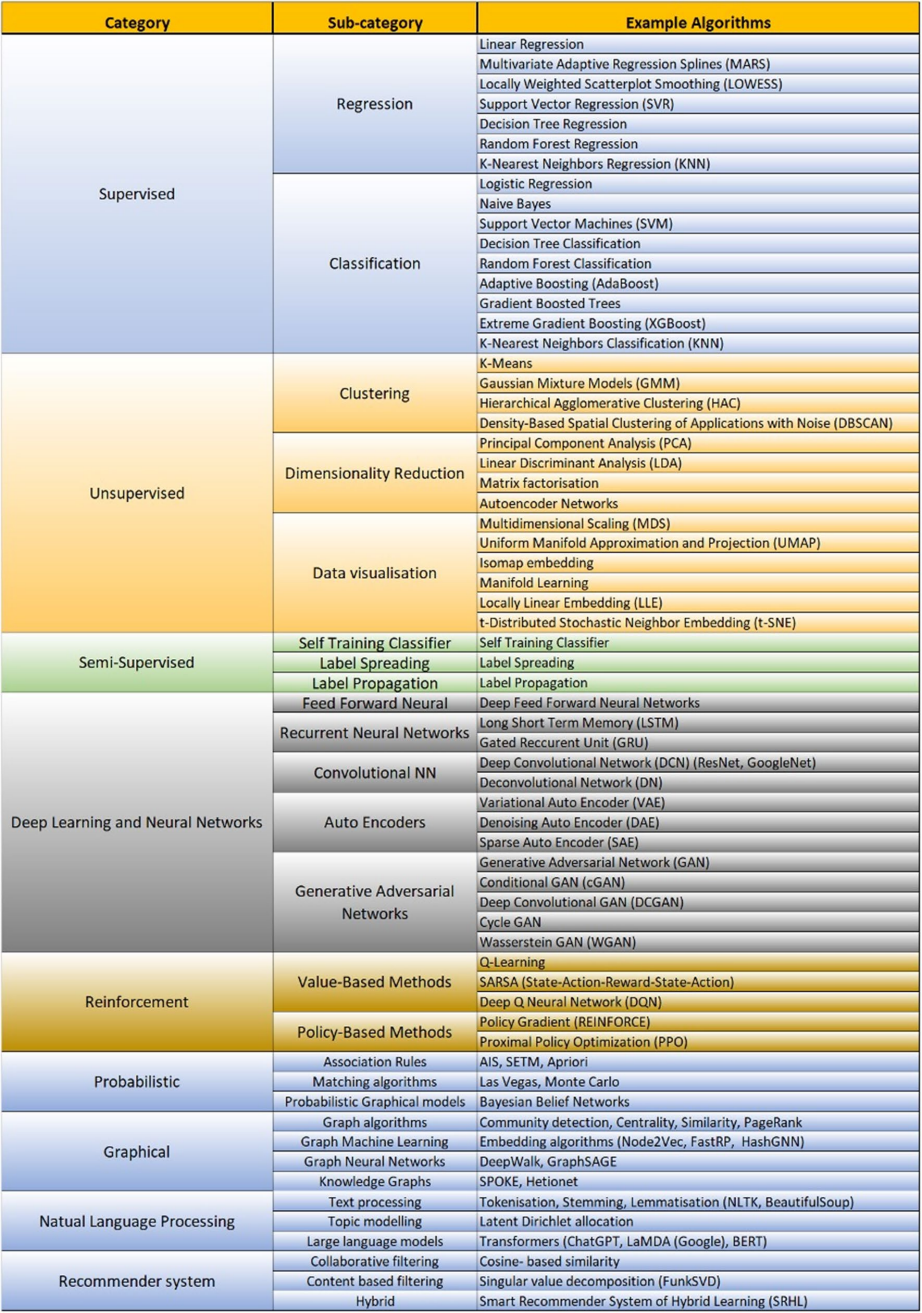
A taxonomy of machine learning algorithms

ML algorithms can be broadly divided into six different categories (SL, UL, semi-supervised learning, SSL, DL, RL, and Other) with numerous classes within each (Fig. 1). It is important to note that the boundaries for many of these algorithms are fluid and each algorithm can potentially be classified under multiple subgroups. Figure 1 is, therefore, used as much for illustration of the breadth and diversity of machine learning algorithms as it is for classification purposes.

- Supervised learning (SL) is used when predicting specified outcomes from a collection of predictors. The data require labelling of the outcome, and training with the labelled data. SL creates an automated system that determines whether items of interest (e.g., patient clinical features) belong to a specific class (e.g., presence or absence of disease).
- Unsupervised learning (UL) is used when only data without specific outcomes and labelling is available. The similarity of observations within the data is assessed to divide the data into distinct groups without previous labels.
- Semi-supervised learning (SSL) involves a mixture of SL and UL. In many situations, the cost of labelling can be relatively high due to dependency on qualified human experts. Consequently, when labels are missing for most of the data but present in a few, semi-supervised methods can be used for model construction.
- Deep learning (DL) models and neural networks are used with both labelled and unlabelled datasets (e.g., image analysis with labelled data or clustering with unlabelled data). In this sense, neural networks are a sub-domain of both SL and UL, but with a unique structure and approach to minimising error [19]. DL models have between several and many thousands of hidden layers, with each layer learning new features from the previous layers [19].
- Reinforcement learning (RL) algorithms are trained to learn the best policy from a set of possible actions by interacting with its environment. The algorithm, known as the agent, receives feedback in the form or rewards and punishments which are based on the changes to the environment resulting from the previous action. The choice of each future action is based on both the immediate rewards from an action, and the potential for future rewards given that immediate action. In medicine, the actions might be a set of possible treatment options, the environment the physiological function of the patient, and the rewards the health status of the patient.
- Other models are algorithms that do not fit easily into other categories include probabilistic models, graphical models, natural language processing, and recommender systems.

These six sub-categories of machine learning algorithms are each described in further detail below.

## Supervised learning

SL, the most widely used form of ML [13], is an umbrella term for algorithms trained on labelled data for the purposes of prediction, where the ground truth of the outcome (label) being predicted is known for each subject in the dataset, having been determined by clinical experts in the field. The learning aspect of SL algorithms is their improvement in accuracy, which is gained from experiencing new input and output data-pairs. An important consideration when training SL algorithms is the requirement for labelled data which incurs considerable time and/or cost, especially for large datasets which is desirable for their training, especially DL algorithms using image data [20]. Relevant examples of expert labelling in geriatric care include referable diabetic retinopathy using fundus images previously graded by medical experts [21], detecting endometrial cancer using segmented CT images of the endometrium and uterus that were previously labelled by radiologists [22], predicting 12-month post-hospital admission mortality using clinical characteristics and laboratory values with verification of vital status using the Australian national death registry [23], and using data on limb movement during timed up-and-go tests to predict fallers and non-fallers [24].

SL algorithms generally consist of developing an explicitly parameterised function that can be used to approximate the outcome for a given set of input data [13]. These functions are sub-divided into classifiers, when the outcome being predicted is binary or categorical, such as in disease prediction, and regressors, when the outcome is continuous, for example length-of-stay or blood pressure. SL algorithms borrow methods from many scientific fields including mathematics, computer science, and optimization theory. Since each algorithm type has its own various strengths and weaknesses, a process known as model selection commonly takes place during training, where multiple algorithms from a family of suitable models are trialled within the same study [13]. In addition, ensemble averaging can be used to combine the results from either different algorithms or the results from a single algorithm type but with a selection of different hyperparameters or datasets. Final classification of the outcome is then based on the majority predicted class across the various models.

The breadth of SL algorithms is reflected by the number of different SL classifiers and regressors supported by the popular scikit-learn library (version 1.1.2) in the Open access Python software [25]. Regressors include linear models (Ordinary Least squares (OLS), LASSO, ridge regression), linear and quadratic discriminant analysis, kernel ridge regression, support vector machines (classifiers and regressors), stochastic gradient descent, nearest neighbors, gaussian processes, cross-decomposition, naïve bayes, decision trees, ensemble methods, multiclass and multioutput algorithms, feature selection, isotonic regression, probability calibration and neural network models. Each model also has a range of hyperparameters that are tuned (varied) to optimise learning and improve accuracy. During this training process, it is imperative to maintain a balance between under and over-fitting of the model, known as the bias-variance trade-off. Over-fitting of the model is indicated if its performance is surprisingly low when assessed on a validation set compared to the accuracy for the training data. This is typically prevented with cross-validation procedures which provide ‘out of sample’ estimates of performance during the training process itself, and with the use of regularisation to limit the number of included features [26]. This data-driven approach to model selection contrasts with traditional domain-driven and statistical approaches in which features are hand-selected, typically assumed to have linear effects and final model selection is based on statistical inference. Unfortunately, this often results in poor generalisability to different datasets and may also miss novel patterns and features [27].

## Unsupervised learning

UL algorithms have several different uses, all of which relate to finding hidden structure in collections of unlabelled data where there is no pre-defined specific disease or health outcome of interest to predict. Python's scikit Learn library includes gaussian mixture modelling, manifold learning, clustering, bi-clustering and co-clustering, covariance estimation, novelty and outlier detection, density estimation, and unsupervised neural network models [28]. UL algorithms can also be broadly classified into those used for clustering, dimensionality reduction, and data visualisation.

### Clustering

Clustering is the most common task within UL and in medicine is commonly used to identify either disease phenotypes (the clustering of patients into meaningful groups based on their various diseases) or clinical phenotypes (groups of patients that tend to share the same set of clinical characteristics). Following the clustering process, each identified group is subjectively labelled according to those features used for the clustering that also differentiate the groups. A benefit of UL algorithms in comparison to SL is their ability to automatically identify patterns and dependencies in the data to provide a compact and general representation of the data [27]. The process of clustering also generates newly learned (engineered) features including cluster membership and other measures of similarity including the patient-to-centroid cluster distances, providing important additional information on patient similarity beyond the raw features [29].

The broad mathematical approaches underlying the clustering algorithms provides a basis for algorithm categorisation as being either partition-based, hierarchical-based, model-based, density-based or graph-based [30, 31]. Table 1 describes the features for each of these five major categories, with examples of their use in older clinical populations. Other algorithms include those based on fuzzy-theory (labels exist on a continuum between 0 and 1), statistical distributions (clustering according to underlying statistical distributions), grid-based (original data is transformed into grids) [32], and fractal theory (similarity based on the invariance when viewed using different scales) [31]. Clustering algorithms may also be simply categorised as either simply traditional or modern [31].

**Table 1:** Cluster-based algorithms used in healthcare research [30, 31]

Taxonomy and algorithm	Examples in healthcare research
**Partitioning**	
**Algorithms:** K-means, K-medoids, Partitioning Around Medoid (PAM), Clustering Large Applications (CLARA) [185], Fuzzy C-Means, Fuzzy Compactness and Separation (FCS), Latent Dirichlet Analysis (LDA), Latent Class Analysis (LCA), Combinatorial k-means **Specific features:** K-means Subjects are continually re-allocated to the closest centroid until centroids are fixed [186] Fuzzy c-means Like K-means, but membership probabilities for each cluster are calculated Combinatorial k-means (Determines optimal feature-set for maximal class separation.) K-medoids (good for outliers.) CLARA (good for scalability due to use of data sampling.)	**K-means:** Use of LCA and K-means to Identify complex patient profiles [187] **K-medoids:** Plasma biomarkers identified adults at risk of Alzheimer's disease and related dementias [188] **PAM:** Identifying subgroups among Home Health patients with heart failure [189] **CLARA:** Identifying ED patient subgroups [190] **Fuzzy C-Means:** Polypharmacy patterns in multimorbid older persons with CVD [191] **Fuzzy Compactness and Separation (FCS):** Clustering of fMRI data [192] **LCA:** Clustering of multimorbidity patterns to examine risk of developing dementia [193] **LDA:** Examining trends in Alzheimer's Disease Research using PubMed abstracts [194] **Combinatorial k-means:** Clustering of T2DM [195]
**Hierarchical**	
**Algorithms:** Hierarchical, Balanced Iterative Reducing and Clustering using Hierarchies (BIRCH), Clustering Using REpresentatives (CURE), STatistical INformation Grid-based (STING), Spectral, Affinity propagation **Specific features:** Hierarchical: Each subject is initially considered as being a cluster. Based on similarity (e.g. Euclidean distance in d-dimensional space), the closest clusters are merged and continues until all subjects have been merged into either the pre-specified number of clusters (k), or one cluster [186] BIRCH: Speed, scalability; CURE: Arbitrary shapes. Spectral: Performs dimensionality reduction before clustering based on the similarity matrix which describes the similarity between each pair of data points	**Hierarchical:** Comparison of multimorbidity patterns in Hong Kong and Zurich using hierarchical agglomerative clustering [196] **BIRCH:** Ability to detect outlier clusters of depressed patients and polypharmacy patients not detectable using regression methods [197] **CURE:** CURE-SMOTE – a hybrid algorithm for feature selection, parameter optimization and synthetic minority oversampling technique (SMOTE) based on random forests [198] **STING:** Useful for mining of geospatial data [199] **Spectral:** Clustering high-dimensional data via feature selection [200] **Affinity propagation:** Parallel clustering algorithm for large-scale biological data sets [201]
**Model-based**	
**Algorithms:** Gaussian Mixture Model, (GMM), Expectation–Maximisation, (EM), Dirichlet Mixture Model, (DMM), CLARANS, Self Organisng Map (SOM), Adaptive Resonance Theory, (ART) **Specific features:** Integrates background knowledge into gene expression, interactomes, and sequences. Models are an oversimplification since assumptions may be false and then results are inaccurate	**GMM, EM, DMM, CLARANS, DBSCAN:** Clustering compositional data using Dirichlet mixture model [185]
**Density-based** **Algorithms:** Density-Based Spatial Clustering of Applications with Noise (DBSCAN) [202], Ordering Points To Identify Clustering Structure, (OPTICS), Mean-shift **Specific features:** DBSCAN regards clusters as dense regions of objects in space that are separated by regions of low density. No pre-defined K required	**DBSCAN:** Machine Learning Technology-Based Heart Disease Detection Models [203] **DBSCAN, OPTICS, Mean-shift, + Affinity Propagation, BIRCH:** Exploring Unsupervised Machine Learning Classification Methods for Physiological Stress Detection [204]
**Graph-theory**	
**Algorithms:** Minimum Spanning Tree** (**MST), Molecular Complex Detection (MCODE), Super Pragmatic Clustering (SPC), Restricted Neighbourhood Search Clustering (RNSC), Markov Clustering (MCL) **Specific features:** Often used for protein–protein interaction networks. Sensitive to user-defined parameter values, often slow	**MST:** Reliance on Visual Input for Balance Skill Transfer in Older Adults: EEG Connectome Analysis Using Minimal Spanning Tree [205] **MST:** Machine-Learning Classifier for Patients with Major Depressive Disorder: Multi-feature Approach Based on a High-Order Minimum Spanning Tree Functional Brain Network [206]

Model selection for UL includes the consideration of data type (categorical, binary, continuous), missingness, computing power, and the shape of the underlying clusters or manifolds. A common problem with health data and especially large electronic medical record (EMR) data is the existence of missing data which presents problems for many optimisation algorithms including K-Means, expectation–maximization (EM), and hierarchical clustering. In contrast, probabilistic clustering models, including gaussian mixture models (GMM) handle missing data efficiently under certain assumptions [33]. Clustering algorithms also vary considerably in their demands for computation, with so-called greedier algorithms trialling all possible solutions and requiring more computing power and time.

#### Measures of clustering accuracy

The aim of phenotyping is to cluster individuals into groups with similar features, with the optimal number of clusters determined by the lowest number of clusters that maximises intra-group homogeneity, inter-group heterogeneity and provides consistent clinical interpretation. Clustering “accuracy” can be assessed using internal or external measures according to whether the clusters are not, or are, known in advance. Internal accuracy measures include Dunn's validation Index, the Silhouette Index, and the Elbow method. External measures include the Jaccard Index (JI), the Adjusted Rank Index (ARI), the Fowlkes Mallows Index and the Normalised Mutual Information [34]. In a comparison of methods using artificially simulated datasets, Spectral clustering outperformed other algorithms including hierarchical, K-means, CLARA, HCMODEL, OPTICS, DBSCAN and Expectation–Maximisation. Higher accuracy was obtained for datasets with approximately 50 normally distributed features [34], whereas performance for other algorithms were comparable for smaller datasets. Clustering accuracy also depends on the hyperparameters selected, with default values usually being suboptimal. If weak cluster solutions are obtained, using a random selection for each hyperparameter often improves performance beyond the default values [34].

#### Dimensionality reduction

Dimensionality reduction is used in both SL and UL settings and typically involves either removing non-useful features (feature selection used in SL) or transforming features to new features (feature transformation used in UL). Feature selection can improve performance when training SL algorithms on medical datasets with high-dimensionality, and sparse, sometimes correlated features. For example, in predicting the risk of mortality after ST-elevation myocardial infarction in older Asian patients, ML models constructed using reduced sets of features demonstrated higher area under the curve (AUC) values compared to ML models developed using a complete set of features. This included logistic regression (0.91 vs. 0.83), random forests (0.91 vs. 0.89), XGBoost (0.89 vs. 0.89) and support vector machine (0.91 vs. 0.87) [35]. Since DL algorithms perform their own indirect feature selection to extract important information, their performance remained higher when all features were retained (AUC = 0.93 vs. 0.91) [35], although prior feature selection will still reduce computing time and costs.

#### Dimensionality reduction using feature selection

Here, the most predictive features are selected prior to full model training. Feature Importance, which ranks each feature in the dataset is generally generated using a random forest algorithm for the purpose of feature selection, although all tree-based algorithms can generate feature importance. Sequential backwards selection is a technique used to remove less informative features. Using the joint application of voxel-based morphometry (VBM) and surface-based morphometry (SBM), a total or 778 features morphological were extracted (170 VBM and 608 SBM) to identify progressive mild cognitive impairment (pMCI) patients that might progress to Alzheimer’s disease [36]. To exclude irrelevant features for training and to efficiently reduce the dimensionality of the dataset and solution space, unsupervised learning algorithms were used including LASSO and gradient boosting decision trees (GBDT) for feature selection. Model classification performance was evaluated using 50 random splits of the training data in addition to a fivefold inner loop cross-validation. A radiomics score (RS) was generated using the best SVM algorithm. The accuracy of the new model with features selected from a combination of the RS, Alzheimer’s Disease Assessment Scale (ADAS) and apolipoprotein E (APOE4), achieved an AUC of 0.867, higher than that achieved for a RS model alone (0.828), or using the ADAS (0.720) and APOE4 (0.591) alone.

#### Dimensionality reduction using feature transformation

Here, the primary objective is to convert high-dimensional data to low-dimensional data (many features to only a few features) without aiming to also retain the original format of the features.

- Principal components analysis (PCA) aims to maximise the explained variance of the original data by generating a new transformed set of variables called principal components. The first principal component retains the largest percentage of information (explained variance in the data) and each succeeding component gradually less information. The user can choose how many components are retained, although the number required to retain 80% of the original variance is a common benchmark. The method is useful in different settings including when latent features are driving the patterns in data, for visualising high-dimensional data in two dimensions, reducing noise, and for pre-processing data to improve the SL algorithm performance. PCA has been used when identifying patterns related to disability vs. autonomy in older adults [37], identifying multimorbidity patterns in nursing home residents [38], and in extracting comorbidity patterns which were then used in predicting mortality [39].
- Random projection is a similar, but more powerful technique to PCA and useful when there are too many features for PCA. Like PCA, the user can choose the number of components to retain, or a value for epsilon (ε), the level of error, can be pre-set. Random projection has been used to predict metastasis in patients with gastric cancer based on CT abdomen imaging [40].
- Independent component analysis (ICA), like PCA and random projection, tries to isolate a sub-group of independent features within the data by finding the features with the smallest correlation with other features.
- Linear Discriminant analysis (LDA) performs dimensionality reduction with transformation of features in a way that also aims to ensure that clusters in the transformed (lower) dimensional space are also well-separated according to labelled classes. The maximum number of transformed dimensions is one fewer than the number of labelled classes. Therefore, for two labelled classes, only one transformed feature can be created. For three classes, two dimensions are created, allowing a scatter plot of the two dimensions with colour coding by class. LDA also provides a prediction model with the coefficients derived from training data. When comparing the oral microbiome profiles of athletes after they followed a low carbohydrate high fat diet and two different consistent carbohydrate-based diets, LDA but not PCA, captured changes in the relative abundance of specific oral microbiota taxa [41]. LDA was used to distinguish profiles of cognitive decline using gender, school years, age, occupational status, and mood based on the geriatric depression scale. Classification into one of three profiles was achieved with an accuracy of 65.9% and Kappa statistic for agreement beyond chance of 0.282 [42].

#### Data visualisation

Understanding the underlying structure of high-dimensional datasets is difficult and reduction to either two- or three-dimensional space is required for visualisation. Although this can be achieved using linear mapping techniques such as PCA, LDA, and others, using these methods may miss important non-linear structure in the data. Data visualisation techniques therefore typically combine manifold learning and Isometric mapping [43]. Manifold learning is a non-linear approach to dimensionality reduction and can be used to learn the underlying data manifold (shape). This is followed by mapping into a lower dimensional space. The primary aim is more accurate visualisation of the underlying nature of the data and extraction of any underlying patterns and biological insights.

t-SNE (t-distributed Stochastic Neighbourhood Embedding) aims to preserve local data proximity and will tend to extract clustered local groups of samples. The first transformation step involves creation of a manifold approximation of the raw data to reduce dimensionality using k-means clustering. The second step is to transform the manifold into new coordinates for visualisation that are optimised to preserve the local distances in the raw data. Data point affinities in the original space are represented by Gaussian joint probabilities and the affinities in the embedded space are represented by Student’s t-distributions. This allows t-SNE to be particularly sensitive to local structure and has a few other advantages over existing techniques, namely:

- Revealing multiple, different, manifolds or clusters.
- Revealing structure at many different scales in a single mapping.
- Reducing the tendency of clustering to clump points together at the centre.

**Fig. 2:**
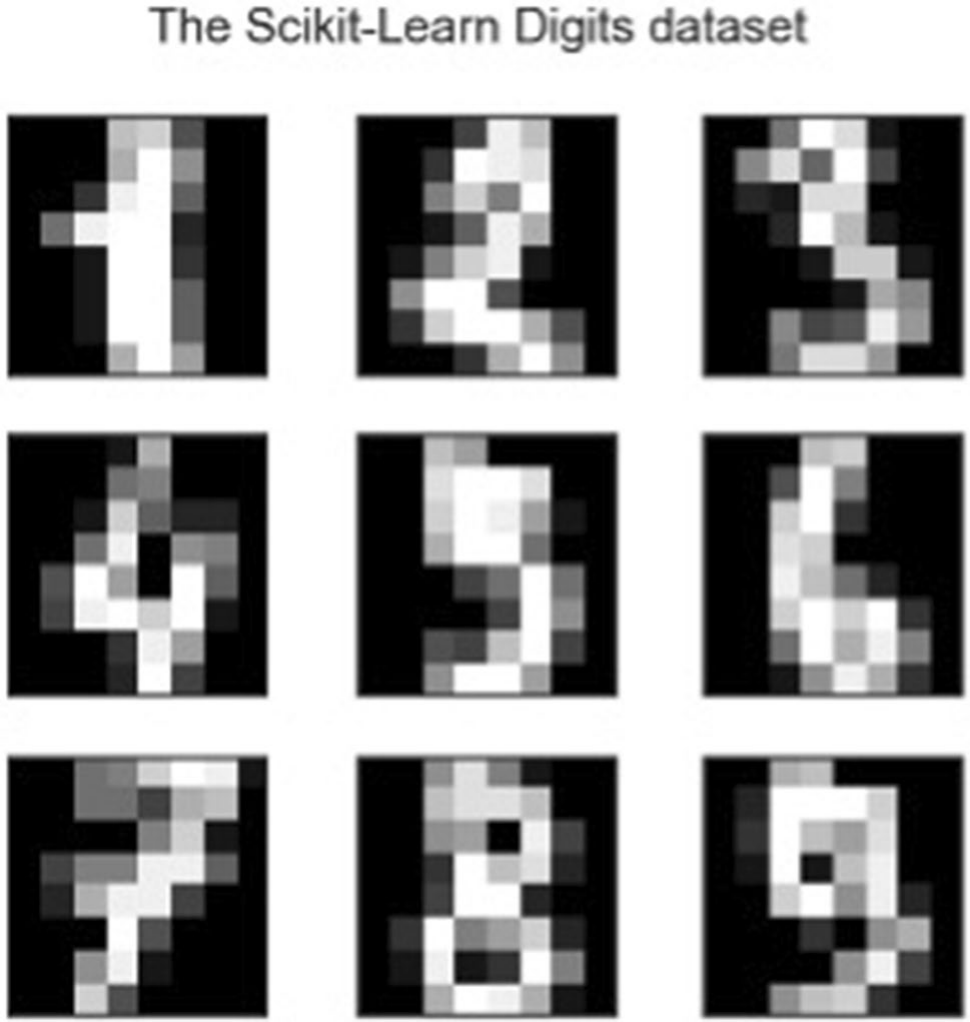
Example data from the scikit-learn digits dataset (*N* = 1797 datapoints). Each digit is described using 64 features which represent the shade (as a numeric value) of each of the 8 × 8 = 64 Gray-scale pixels used to describe a zero to 9 digit

**Fig. 3:**
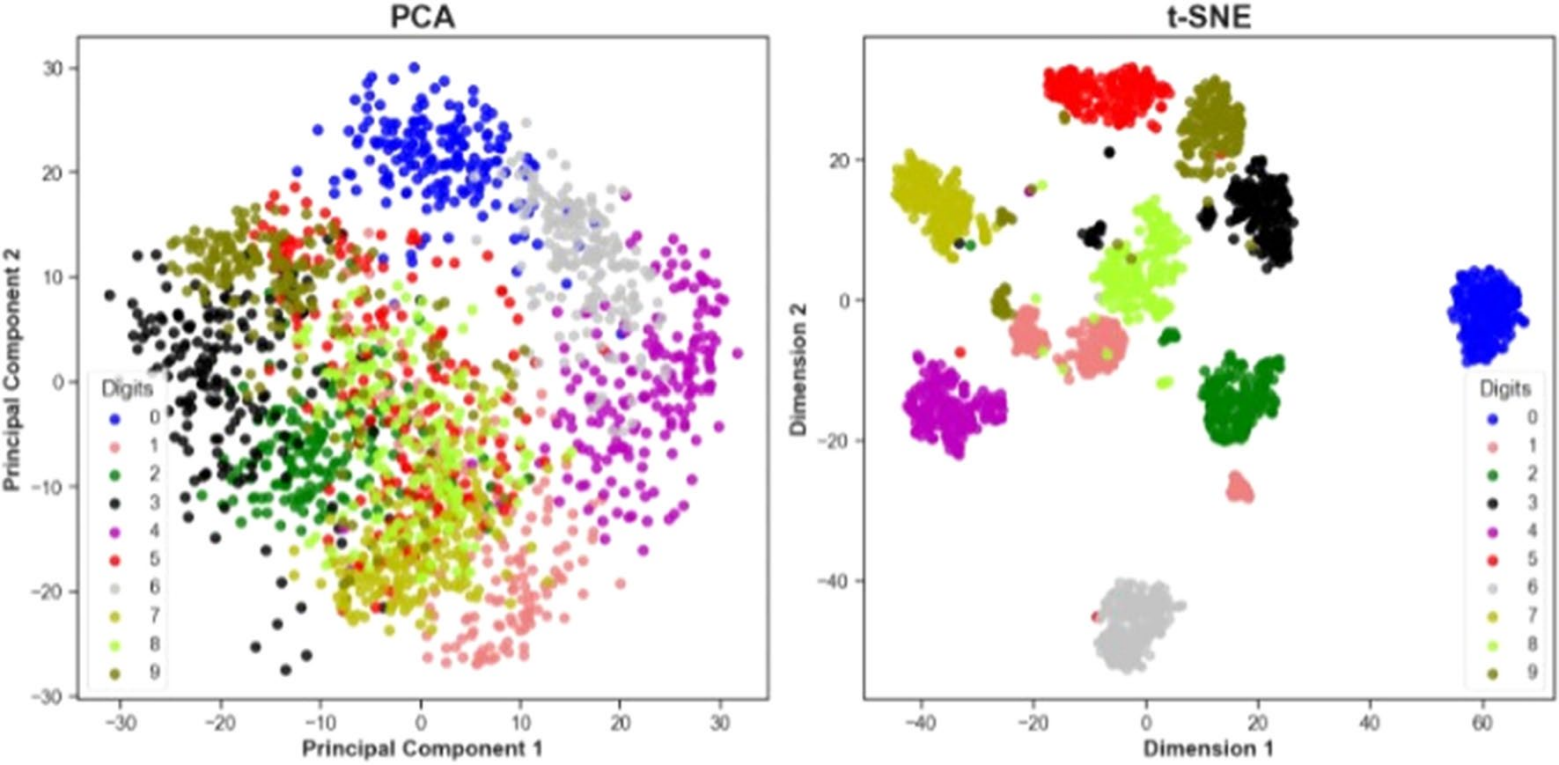
Clustering of the scikit-learn digits dataset using principal components analysis (PCA) and t-stochastic neighbourhood embedding (t-SNE) (*n* = 1797). The scikit-learn digits dataset consists of 1797 datapoints and 64 features, with each feature representing the shade of pixel in an 8 × 8 = 64-pixel image of a digit (0 to 9). Visual mapping of the features into 2-dimensional space using t-SNE was more successful than PCA, reflecting the greater ability to retain the feature similarity of the 64 features (numeric values for a pixel colour) that describe a single digit. A trade-off for this improved accuracy is the difference in algorithm complexity and the corresponding time for execution. The PCA analysis was performed in 0.015 s, whilst the t-SNE analysis took 41.09 s

Figure 2 and Fig. 3 illustrate these capabilities using the digits dataset available in Python's scikit-learn library. The dataset consists of 1797 records with 64 features, each feature representing the pixel density of the 8 × 8-pixel images of a digit from 0 through to 9 (Fig. 2). Clustering using PCA and using the t-SNE algorithm separately results in the clusters shown in Fig. 3, with much a clearer separation produced by t-SNE than for PCA.

Many other mapping techniques that exist are often extensions of previous techniques, designed to improve mapping by retaining the key similarities and clustering of the original data. Different mapping equations and the focus on different distances provide subtle differences in how the original data structure is preserved when mapped. Isometric mapping (Isomap) preserves geodesic distances, Multidimensional scaling aims to preserve all distances, spectral embedding, and locally linear embedding (LLE) preserves local distances, and Hessian Eigen-mapping preserves local linear structure. Each method has its advantages and disadvantages. Isomap, LLE, and their variants are best suited to unfold a single continuous low-dimensional manifold. On the other hand, t-SNE can reveal data that lie in multiple, different, manifolds or clusters. Isomap is an extension of multidimensional scaling and densMAP is an extension of t-SNE and Uniform Manifold Approximation and Projection (UMAP) [44] better capturing the original density of the local data in the two- or three-dimensional space. An ability to group samples based on the local structure can be beneficial to visually disentangle a dataset that comprises several manifolds at once. UMAP typically results in more distinct and more compact clusters than t-SNE.

#### Examples of clustering in older populations

##### Responses to visual stimuli

Mapping clusters with t-SNE was used as a visualisation tool to investigate possible differences in algorithm accuracy for classifying responses to four different visual stimuli tasks using a brain-computer interface [45]. The t-SNE mapping showed differences in the separation of the four response clusters between younger and older age-groups, which suggested differences in the accuracy of the classification algorithm between the two age-groups.

##### Clustering intrinsic capacity

Patterns of intrinsic capacity (IC), a novel patient-centred functional measure in older patients [46] was examined using K-means and t-SNE. Intrinsic capacity was measured using 13 variables to measure locomotion (4-m walk-speed, balance, chair-rise, grip strength), vitality (body mass index, unintentional weight-loss, swallowing), cognition (mini-mental state examination), psychological (depressive symptoms, sleep quality, satisfaction with life), and sensory capacity (hearing and vision). The total IC score was calculated as the sum of the five domain scores. The K-means analysis identified 5 clusters of intrinsic capacity, whilst using t-SNE allowed mapping of the high-dimensional space (13 features) into a low (2-dimensional) space with the clear presence of five distinct clusters which varied in the level and type of impairment.

## Semi-supervised learning

Datasets for which only a relatively small proportion of labelled samples exist often occurs with medical data since the precise labelling of individual samples can be either expensive or time-consuming or both. Several SSL methods can overcome these limitations including weakly supervised learning based on weak annotation, self-supervised learning based on representation learning, and semi-supervised learning based on limited annotation. These new methods have led to a new wave of automatic labelling and analysis targeted at annotation efficiency [47]. Scikit Learn includes self-training and label propagation SSL estimators designed to capture the shape of the underlying data distribution. Since labelled ground truths are not always accurate, SSL approaches can even outperform SL approaches.

### Self-training

In self-training, a meta-estimator approach is used, involving the use of different supervised classifiers. The supervised classifier then learns from unlabelled data by iteratively predicting pseudo-labels for the unlabelled data and adding them to the training set. The number of available algorithms in self-training learners is dictated by the number of suitable supervised classifiers that one might consider using for the task of prediction.

### Label propagation

Label propagation and label spreading both work by constructing a similarity graph over all items in the input dataset. A normalised graph laplacian matrix is constructed in the same manner as for spectral clustering. The algorithm identifies areas where the structure of unlabelled observations is consistent with the labelled classes, allowing class labels to be propagated to the unlabelled observations.

### Label spreading

These algorithms are like Label Propagation algorithms but use the affinity matrix based on the normalized graph Laplacian and soft clamping across the labels. In soft clamping, previously defined labels are allowed to be changed (with a pre-defined level of randomness).

#### Examples in healthcare

##### Predicting urinary tract infections

The indication of a urinary tract infection (UTI) based on urinary analysis is an important factor for clinicians to initiate antibiotic prescribing. SSL based on label-propagation was used to predict urinary analysis results for UTI with improved performance compared to expert-based labelling. A clinical decision support (CDS) tool was then developed using the SSL algorithm with an accuracy and negative predictive value of 85.24% and 87.46% respectively, sufficiently high to potentially reduce inappropriate antibiotic prescribing [48].

##### Predicting cancer progression

SSL was used to determine true clinical event times such as time to cancer progression that are important for personalised prediction of risk and prescribing, but which are not readily available in large EHR datasets [49].

##### Step-up therapy for rheumatoid arthritis

SSL outperformed SL models in identifying rheumatoid arthritis patients that might move from first-line therapy to step-up therapy (disease-modifying antirheumatic drugs or targeted synthetic disease-modifying antirheumatic drugs) within 1 year. Five groups of features were extracted from an administrative claims database: demographics, medications, diagnoses, provider characteristics, and procedures. The semi-supervised approach had significantly higher F-measure (65 vs. 42%; *p* < 0.01), precision (51 vs. 33%; *p* < 0.01), and recall (89 vs. 59%; *p* < 0.01) than the supervised approach. This study showed that SL approaches are not necessarily an optimal option for clinical decisions regarding step-up therapy. Specifically, the negative class labels in step-up therapy datasets are not always a reliable indicator that step-up therapy was not required, because the costs and risks associated with higher line therapy impact the objective decision making of patients and providers.

## Artificial neural networks and deep learning

Despite ML dating back more than 80 years, and DL only became widely accepted as being a viable form of AI in 2012 [50], DL is now considered the future of ML, providing the essential capability for developing human intelligence comparable systems for processing vision, speech and many other complex inputs. In healthcare, DL is being applied to an ever-increasing range of problems for which considerably improved prediction beyond standard scoring routines is possible.

Artificial neural network (ANN) algorithms involve an architecture of interconnected layers, each with a pre-defined number of units that compute some simple parameterized function of its inputs. DL involves the use of ANNs with many layers (typically 5 to 1000), each of which has a particular function, and which progressively detect new features that are learned representations of the input data. As such, the input variables for each layer are not observational input variables but are complex intermediary solutions. Utilising gradient-based optimization, the parameters throughout the network are adjusted according to the layer's previous output errors. Exploiting parallel processing architectures and graphic processing units such as those used for video gaming it is possible to build networks with potentially billions of parameters. In this way, they can model highly complex, non-transparent (e.g., mathematically non-linear) relationships between the input and corresponding output variables [51]. Typically trained on very large collections of images, videos, and speech samples, such systems have yielded major improvements in performance over previous approaches in computer vision and speech recognition [13]. DL is also an important component of UL algorithms. For example, a patient’s full EMR can be presented in a low two-dimensional space using a UMAP (Uniform Manifold Approximation and Projection) [52]. The major limitation of ANNs and DL is their requirement for datasets in the order of several 10,000 data points (subjects) to be successful. In older patient populations, ANNs and DL have been successfully used for predicting depression [53], hospital length of stay [54, 55], sarcopenia [56], and falls [57, 58].

### Feed-forward

Deep feed-forward networks, also often called neural networks or multilayer perceptrons (MLPs), form the basis of many important commercial applications, including the convolutional neural networks used in image recognition. They are also a conceptual stepping stone on the path to recurrent networks, which power many natural-language applications [59]. The goal of feed-forward neural networks is function approximation in which the function *y* = *f*(*x*) is approximated and parameters theta (θ) are learned to provide *y* = *f*(*x*,θ) which is the best approximation for *y* = *f*(*x*). The models are called feed-forward because information flows through the function being evaluated from *x*, through the intermediate computations used to define *f*, and finally to the output *y*. There are no feedback connections in which outputs of the model are fed back into itself as in other DL models. When feed-forward neural networks are extended to include feedback connections, they are called recurrent neural networks.

### Recurrent neural networks

Recurrent neural networks (RNNs) are temporal models that are explicitly multivariate and sequential with an ability to handle time-series data. In healthcare, RNNs allow the temporal dynamic relationships between risk factors for a patient to be integrated into the risk assessment. In a comparison of four different DL architectures applied to the UK General Practice Research Database and linked hospital episodes for over four million patients, RNN was the most suitable architecture due to the temporal nature of EHRs [52]. Application of RNNs in older patients include the assessment of gait patterns [60], and the identification of patients with dementia potentially benefitting from palliative care interventions [61].

RNNs are also commonly used in analysing image data. For example, different output layers from a convolutional neural network (CNN) are generated by focusing on different image areas. From the series of extracted images, an RNN labels the different features of the image such as the number of objects in the image, the type of object, and the background of the image. The RNN then uses this information to generate an appropriate text caption for the original image such as “A bird flying over water” [13].

#### Long short-term memory RNNs

An early limitation of RNNs was their absence of a strong memory and ability to exploit long-distance interactions and correlations. This problem of dealing with long-range dependencies was overcome with the development of RNNs including a long short-term memory (LSTM) hidden unit that remembers the activation patterns of hidden layers. This allows significant events from the distant past to be recalled and unimportant events to be forgotten when making current predictions [62]. Within the context of healthcare, LSTM networks retain the sequential information from patient histories making them especially suitable for long-term forecasting using EHR data. RNNs with LSTM were successfully applied to EHR data to predict future disease diagnoses based on the use of only 13 frequently but irregularly sampled clinical measures with episodes varying in length from 12 h to several months [63]. Data included diastolic and systolic blood pressure, peripheral capillary refill rate, end-tidal CO_2_, fraction of inspired O_2_, Glasgow Coma scale, serum glucose concentrations, heart rate, pH, respiratory rate, blood oxygen saturation, body temperature, and urine output.

RNN with LSTM algorithms using time-series data has provided important benefits in diagnosis and disease management for older patient population in numerous different healthcare domains. Applications include gait analysis [64], discriminating hand-movements [65], adherence to technology-based cognitive training [66], differentiation between stroke and healthy individuals using data on unhindered activities of daily living collected using wearables [67], sleep-stage classification from photoplethysmography [68], improving the prediction of Alzheimer’s disease using medical domain knowledge [69], predicting Alzheimer's disease progression [70], detecting vertebral fractures on CT scans [71], and determining life-expectancy [51], an important patient-reported outcome measure for the older population [51]. In another study, a CNN combined with a LSTM RNN was used to extract spatial and temporal information, respectively, and achieve a 100% accuracy in detection of fallers (*n* = 327/327) and 99.73% (*n* = 1114/117) accuracy in detection of non-fallers [72].

#### Convolutional neural networks

A convolutional neural network (CNN, or ConvNet) is a specialized type of feed-forward network commonly applied to analyse visual imagery. Images are fed through different layers that transform (convolute) the images into new feature sets. Relatively little pre-processing is involved with data-driven automated learning used to filter images rather than using hand engineered feature selection which relies on prior knowledge and human intervention. Recently, tridimensional neural networks (incorporating image width, depth, and resolution) have been developed that improve accuracy and considerably reduce the required number of CNN parameters (often ~ billions). CNNs are also a core initial component of the architectures required to tackle computer gaming problems which typically involve high-dimensional sensory inputs combined with the need to also perform actions. Combined with reinforcement learning, CNNs have been used to develop intelligent agents capable of learning to excel at a diverse array of challenging tasks [73]. CNN’s have been investigated in older patient populations to diagnose dyspnoea with chest X-ray [74], ischaemic stroke [75, 76], and cerebral atrophy using magnetic resonance imaging [77]. In an effort to reduce the subjective assessment of knee radiographs and to improve workload efficiency, an automated approach using CNNs was trialled to assess Kellgren and Lawrence (K&L) grading of osteoarthritis severity amongst 359 participants aged 71–80 from the Hertfordshire cohort study [78]. The diagnostic accuracy was similar from observer-derived K&L scoring and for ML-derived K&L scores, particularly amongst women (AUC = 0.65–0.70 for observer and 0.63–0.68 for ML-derived K&L scores). The study illustrated that automatic K&L scoring from radiographs can be performed using ML which offers potential savings in assessment time, a reduced burden on the radiology workforce, and avoids observer-dependent subjectivity.

#### Autoencoders

Autoencoders are unsupervised feed forward neural networks used for feature extraction and dimensionality reduction, making them comparable to PCA, Linear Discriminant Analysis, Factor analysis, Multidimensional Scaling, Isomap, Local Linear Embedding (LLE), and t-SNE [79]. Autoencoders are typically trained to predict the input as its output, whilst imposing restrictions or bottlenecks to enable learning the most useful information from the data and more appropriate representations. This is achieved by feeding the data through several layers (encoding, hidden and decoding layers) which in addition to providing dimensionality reduction, by including class information in the process, also improve the separation of classes (the outcome labels) according to the new set of features [79]. This reduction in class complexity enables autoencoders to also be used for pre-processing prior to binary classification problems. DL networks in the form of autoencoders have been used to assess the activities of daily living of older adults using electricity consumption data [80], to assess mobility and fall risk [81, 82], and to prevent adverse drug reactions using electronic health records [83].

#### Generative modelling

Generative modelling is a form of synthetic data generation and when performed well can generate new data indistinguishable from the real data. Two modern examples of DL generative modelling algorithms include the variational autoencoder (VAE) and the generative adversarial network (GAN), which are DL-based generative models. GANs were first proposed in 2014 by Goodfellow and developed using a game theoretic scenario in which the generator network competes against an adversary [84]. The two components of the network are the generative model and the discriminative model. The generative model learns a new latent space that is a representation of the actual data, which it can then use to create new examples. The discriminator model takes examples from the domain as input (real or generated) and predicts a binary class label of real or fake (generated) example which comes from the training dataset. The discriminative model is then updated to improve its discrimination, and the generative model is also updated, with the two models being trained together in this adversarial framework. When the discriminator model is being fooled approximately 50% of the time, this indicates that the generator model is generating plausible examples. GANs provide a path to sophisticated domain-specific data augmentation and have achieved photorealistic results for various image synthesis and image-to-image translation problems.

The algorithms used for the generative component of the network utilise common SL and UL algorithms including naïve bayes, Latent Dirichlet Allocation, and the Gaussian Mixture Model (GMM). Naïve Bayes is more typically used as a discriminative model for classification or regression in which the probability of an outcome is calculated for each feature and then combined to provide the class prediction. Used in reverse, it becomes a generative model in which new samples are generated from the probability distributions for new plausible values. A standardized approach called Deep Convolutional Generative Adversarial Networks, or DCGAN led to more stable models. Most GANs today are at least loosely based on the DCGAN architecture and formalized in 2015 [85].

## Reinforcement learning

Reinforcement learning (RL) represents the third major category of ML algorithms after that of SL and UL. Whereas SL learns to predict based on a pre-defined set of rules, RL algorithms learn by interacting with an environment and obtaining feedback on the consequences of its actions in the form of short-term rewards, which assist towards learning the actions that optimise long-term reward [86]. This sequential decision-making problem can be formally formulated as a Markov Decision Process (MDP) given the stochastic nature of an MDP and corresponding uncertainty in outcomes. RL provides a mathematical framework for experience-driven autonomous learning [86], and within medicine the potential to automate treatment decisions that optimise the long-term health of a patient [87]. A difficult challenge when applying RL within healthcare is the appropriate defining of the reward function for each possible change in the patient's physiological state. The assignment of these values will influence long-term rewards (health outcomes) and, therefore, treatment choice [88], even though it is not always obvious what the numerical reward value should be. The adoption of RL in healthcare is still in its early stages with most applications still existing at a research stage but gaining rapid interest. Research applications include optimal treatment policies for diabetes management [89, 90], the simultaneous optimisation of glycaemic control, blood pressure and cardiovascular risk for patients with type 2 diabetes [91], and the treatment of sepsis patients in intensive care units [92].

### The learning paradigm

**Fig. 4:**
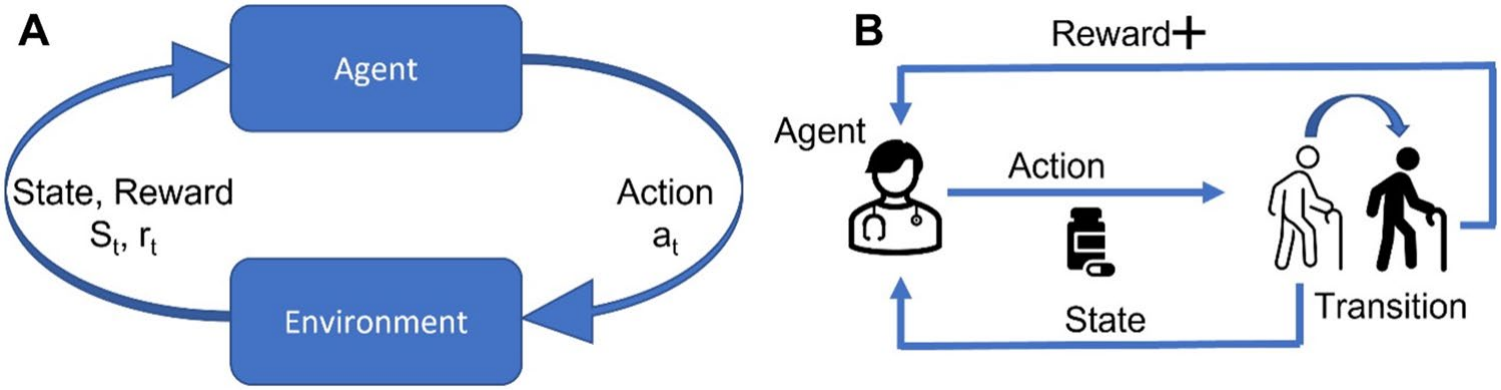
Representations of the reinforcement learning workflow. **A** At a given moment (*t*), the environment provides the agent with a given state and reward signal from its previous action and applies a new action. **B** In the aged care scenario, the agent is the clinician whose actions consist of different treatment recommendations. The environment is the patient that transitions from state to state and provides feedback to the clinician in terms of their given state and the change in their condition (reward). Although the state will change quickly, rewards may be delayed over time

In the RL paradigm, labelled data on what constitutes the correct action is not available, and instead the learner in the RL setting uses a trial and error approach, observing how each action it takes alters the environment (the patient's state) to slowly discover the most likely optimal action [93]. Figure 4 shows different representations of this cyclical learning paradigm, which can be generalised as involving an agent interacting with an environment, receiving feedback in the form of a reward signal, and updating its policy based on this feedback. The goal of the agent is to learn a policy that maximizes the long-term return, which is the sum of the rewards received over time. In the clinical context, the state is a feature-representation of the patient's condition, the action is the treatment decision, the reward is the change in patient's condition, and the environment is the patient and surrounding factors affecting the patient [94]. The reward and state are both fed to the agent to determine the next action.

### Value-based and policy-based reinforcement learning

The two main approaches to solving RL problems are methods based on value functions and methods based on policy search, also known as value-based and policy-based algorithms, respectively. In value-based RL, the focus is on learning a value function (V), or a Q-function, (Q). The value function V evaluates the set of expected long-term rewards for every possible state-action pair in the current state. The Q-function evaluates the set of long-term rewards for each possible state. Once V or Q have converged, the optimal policy can be determined. In policy-based algorithms, the focus is on learning an optimised policy (set of actions) directly. The weights of the algorithm are changed at each iteration and the policy is updated in a way that increases the chances of taking actions resulting in higher rewards. Within these two broad domains of RL, there exists many different sub-categories, each with their own advantages and disadvantages, the optimal choice depending on the data availability and specific problem.

#### Value-based learning approaches

##### Q-learning

Estimating the complete set of Q-values (expected long-term rewards) for each state-action combination is known as Q-learning. When the action-state space is small (only a limited number of states and actions exist), the set of Q-values can be described in an array format with updates to each cell in the array taking place as the learning progresses. Eventually, the Q-values converge to a higher limit and the optimal policy is then determined as being the path of actions in a sequence of states that achieves the highest long-term reward. Q-learning is a form of off-policy batch-mode RL algorithm since learning occurs without needing to apply the policy and generate new samples, and the data has been previously collected and is stored and used in batches for training.

For more complex problems, tabular representations of the *Q*-values for all possible state-action pairs becomes computationally infeasible or even impossible, especially if the states are defined by continuous values rather than discrete vales. In this setting, the use of function approximators to determine the updated *Q* values are used [95]. These include fitted Q-iteration (FQI) models that use SL algorithms to generate linear function approximators, and NN-based models to generate non-linear function approximators (DQN). Both FQI and DQN are value-based off-policy RL models.

##### Fitted Q-iteration

A limitation of Q-learning is that it makes relatively inefficient use of the data and is therefore not ideal for problems where data acquisition is costly. FQI significantly reduces the quantity of data required to learn useful policies and has been used to optimize the treatment of several diseases including human immunodeficiency virus infection and acquired immunodeficiency syndrome, psychiatric disorders, epilepsy, schizophrenia, smoking addiction, and anaemia [96]. The FQI algorithm is a batch mode RL algorithm which iteratively computes a Q-function, a linear approximator for the Q-values [97]. The main feature of FQI lies in the way that it handles the experience. Unlike incremental algorithms, FQI uses the complete set of all possible state-transitions at each step to update the estimation of the optimal Q-function. Although this process involves more computation, it extracts more information from the stored experience and is, therefore, more data-efficient than other RL algorithms. At each step, the algorithm builds a new training set of inputs and target values. The inputs at time-step k, are the values for the current state-action pairs (${s}_{k}^{j}, {a}_{k}^{j}$) for each possible state transition *j* at step k, and the target value ${\widehat{Q}}_{n}$(s, a) is the conditional cumulative reward; the new reward ${r}_{k+1}^{j}$ plus the discounted Q-values from the previous step i.e.,:

1 $${{\widehat{Q}}_{n}\left(\mathrm{s},\mathrm{ a}\right)=r}_{k+1}+ \gamma \cdot \underset{{a}^{\prime}}{\mathrm{max}}{\widehat{Q}}_{n-1 }\left({s}_{k+1}^{j},{a}^{\prime}\right).$$

The term $\underset{\mathit{a^{\prime}}}{\mathrm{max}}{\widehat{Q}}_{n-1 }({s}_{k+1}^{j},{a}^{\prime})$ represents the expected long-term reward from taking the best action in the next state (${s}_{k+1}$). The (*γ*) factor that is applied to this is the discount factor, which determines the relative contribution of previous actions. Equation (1) is known as the Bellman equation.

A SL algorithm, for example regression, extremely randomised trees, or neural networks, is applied to each new dataset to compute the next Q-function of the sequence, producing a sequence of Q-functions which eventually converge. Since all updates are performed offline, approximation of the Q-function can be viewed as a separate SL task.

It is worthwhile to point out that in FQI, no assumptions are made on the ordering of tuples (the set of state, action, reward, next-state observations required for each update). These could correspond to a transition from a single patient admission, or randomly ordered transitions from multiple histories. As such, FQI is particularly suitable for assessing optimal policies based on a sequence of patient admissions, in which most samples are separate patients rather than repeat measures of the same patient.

##### Deep Q-learning

Deep Q-learning (DQN) combines DL with RL, and enabled researchers to overcome many of the previous issues with RL that limited its progress [98]. DQN addresses the issue of instability in function approximation by using experience replay and target networks. Experience replay, which is used to speed up training involves the storing of observations, (sequences of state, action, reward, and next state) in a replay buffer which is sampled randomly during training. Double DQN employs two simultaneous target and evaluation networks and is also used for a more stable learning target and low-variance action-value estimates. The target networks involve a separate set of network parameters being stored whilst training, and only being used for updating periodically, rather than at every training iteration. In the Asynchronous Advantage Actor-Critic (A3C) model, parallel actors and different exploration policies are employed to stabilize training, and experience replay is not utilized. Each of these approaches were incorporated by DeepMinds to successfully train multiple Atari 2600 games [73].

Although the input and target values for approximation of the Q-function are like FQI, only an update of parameters of the neural network (NN) is required to obtain the newly estimated Q-function. Thus, the weights of the NN can simply be updated at each iteration saving considerable time and computing costs, rather than the complete estimation of a new model, which in the case of FQI using extremely randomised trees means building a set of new trees. The deep neural network (DNN) is trained to optimise the Mean Squared Error (MSE) given as:

$$\mathrm{L }(\upphi )=\frac{1}{2}\Vert {V}_{\phi }^{\pi } \left({s}_{t}, {a}_{t}\right)- {y}_{t+1}\Vert$$.^2^

where the target value ${y}_{t+1}=r({s}_{t}, {a}_{t})+ \gamma \cdot \underset{{a}_{t+1}}{\mathrm{max}} {Q}^{\pi }({s}_{t+1}, {a}_{t+1})$

This optimization can be done with various Gradient Descent methods used in DL, such as Stochastic Gradient Descent (SGD). Following this computation, the policy is updated from *π* to *π'* only if the action taken from *π'* resulted in the maximum for the Q-function[95].

DQN allows separate NNs to be trained for each possible action with the output being the probability of taking each action, or a single NN to be trained across all states with the NN weights shared across the various functions, and the output being the probabilities for each action. Advantages of using DQL include its ability to leverage the representational power of DNNs and develop non-linear function approximators. However, with insufficient data volumes, the DQN is not guaranteed to achieve a stable RL policy whereas FQI is guaranteed convergence for many commonly used regressors, including kernel-based methods and decision trees.

##### Policy-based reinforcement learning

In policy-based, or policy-gradient RL, the aim is to find an optimal policy by evaluating the reward trajectories for different policies. To start, a random policy is first applied, and the algorithm interacts with the environment, generating a sample of sequential state-action pairs which form a single trajectory. After several trials and trajectories are collected the rewards for each trajectory are evaluated and the RL policy is updated to increase the chance of visiting highly rewarded trajectories and reduce the chance of visiting lowly rewarded trajectories. New state-action pairs are then generated from the updated policy, which are then again evaluated, and the policy is updated and improves over time by “trial and error”. Although this approach is fine for problems where data generation is performed by simulation such as computer-board games or robot control, for clinical situations, it would be unethical to simply use a “trial-and-error” approach which would be too costly and time-consuming to perform in real-time. In the clinical context, therefore, policy-gradient RL is, therefore, not as popular compared to other RL algorithms.

##### Off-policy versus on-policy methods

Policy-gradient RL algorithms are also described as being on-policy RL, because the same policy is used for the agent to act (acting policy) and to update the value function (updating policy). This contrasts with off-policy where we can improve the policy without needing to generate new samples from the updated policy. Policy-based approaches include dynamic programming, Monte Carlo, and temporal difference models. The choice between off-policy and on-policy methods is influenced by the nature and availability of the data that describes the state-action space. Policy-based methods are more useful if the state-action space is continuous, and evaluating an infinite number of actions or states using value-based methods would be too computationally expensive. However, a drawback of policy-based RL models is the demand for a sufficiently large set of data for training to ensure that the learned policy converges to an optimal one. Policy-based methods also use online learning, in which the learning is happening as the data comes, imposing time constraints that could be impractical if the feedback were too slow. In offline learning with a static retrospective dataset, there is no requirement for direct exploration or environmental interaction during training [99].

##### Actor-critic

**Fig. 5:**
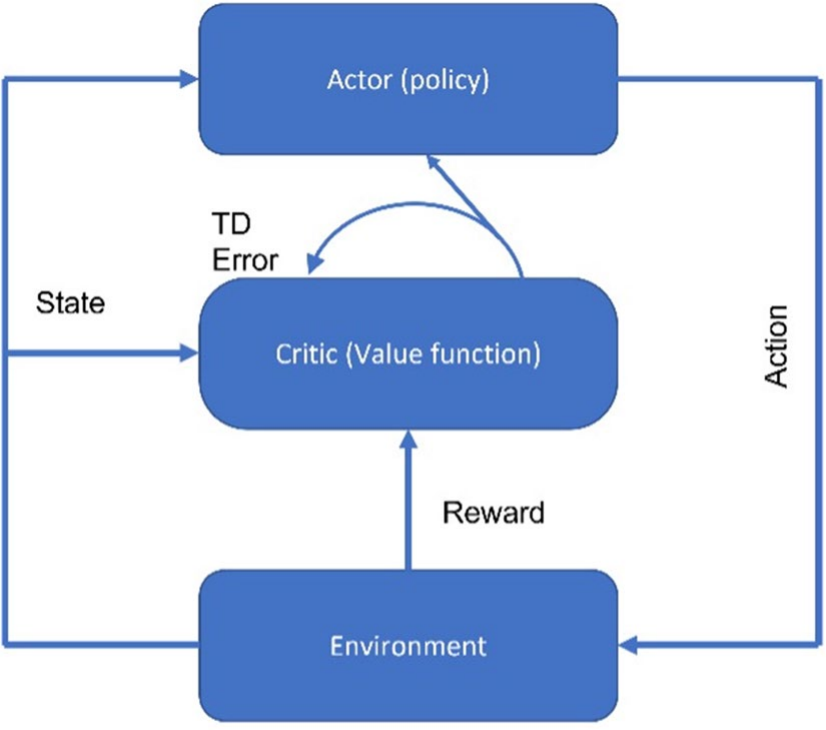
Architecture of the actor-critic network. The actor takes the environment's state as input and produces a probability distribution (policy) by which an action is selected. The critic takes the same state and estimates the value function. The selected action is executed, the next state is generated from the environment and a reward is computed. The next state is forwarded to the critic network to get the value of the next state. The temporal difference (TD) error is then computed using the Bellman equation and used to update the critic network

The hybrid actor-critic approach employs both value functions and policy search. Most health RL studies use off-policy value-based methods including FQI and DQN. These methods necessarily use previously collected data obtained using an existing policy, for example a clinician treating patients that generated a collection of patient states, actions, and outcomes. The goal of the FQI or DQN RL network is to improve upon the clinician policy, by estimating Q-values from actions that originated from a clinician policy. Rather than relying on historical values, the actor-critic RL model uses two separate RL networks within one model, with a policy-based (actor) network and a value-based (critic) network, which is jointly learned with the actor network. State-action pairs are first generated by the DNN in the actor network, and the critic network takes these state values as input to generate a value function V using its own DNN (Fig. 5) [87]. V is then used to update the policy parameters of the actor. V is evaluated as an advantage function describing how much better a specific action is in the current state than the average action in that state.

##### Additional RL architectures

The above section provides a differentiation between the two very broad categories of policy-based learning and value-based learning. Since Q-Learning first appeared in 2003 [100], many modifications of the original architecture have been developed to deal with issues such as efficiency (computing time), convergence, instability, causality, and accurately describing the reward function. Subfields of RL that have arisen to tackle these specific problems include hierarchical RL, recurrent RL, inverse RL [88, 100–102], and causal RL [103, 104]. These and others have been reviewed in recent RL surveys [105].

#### Examples of reinforcement learning in older populations

##### Conversational robots for persons with dementia

RL algorithms were developed to train robots to develop an adaptive conversation strategy and have interactive conversations with persons with dementia [106]. The conversational robots could reply and create follow-up questions designed to distract the subject from using repetitive questioning, a common feature of patients with dementia which is exhausting for caregivers and prevents regular meaningful daily interactions. The RL approach allowed the ability to generate responses that are adapted to the personality and cognitive ability of individual patients and the integration of developer-defined rewards that incorporated the long-term influences of the generated responses. Based on average returns and Q-values, the adaptive action selection using RL performed better than random question selection and demonstrated the potential of conversational social robots to prevent the repetitive questioning behaviours of patients with dementia and stimulating their brain activities.

##### Optimising haemoglobin concentrations in patients undergoing haemodialysis

The response to erythropoiesis-stimulating agents in patients receiving haemodialysis varies widely according to comorbidities, diseases severity, clinical characteristics, and concurrent medications. Achieving stable haemoglobin levels within the target range is, therefore, complex and often requires dose titration. An FQI RL algorithm was used to optimize the use of erythropoiesis-stimulating agents and prevent anaemia. To reduce dimensionality, patient states were defined using a k-means derived group variable based on three variables (endogenous EPO, Cr and Cp) and gender. Actions were drug doses of 0, 0.25, 0.50, 0.70, and 1.0 μg/kg darbepoetin alfa per week. A drug administration policy was learned using both FQI and Q-learning that selected actions at each month according to the observed states. FQI performed substantially better than standard Q-learning algorithms and the standard protocol, increasing the proportion of patients within the targeted range of haemoglobin from 54.5% for the standard protocol to 84.1%, and reducing the required drug by 5.13% in the mean recommended dose from 0.39 to 0.37 μg/kg/week [96]. The temporal variability of haemoglobin concentrations was also significantly lower for FQI than the standard protocol. The study resulted in a clinical decision support tool for clinical evaluation across five haemodialysis centres from three European countries.

##### Optimising catheter ablation during surgery for atrial fibrillation

The success rate of catheter ablation for atrial fibrillation (AF) is currently poor, ~ 50% after 2 years. Deep Q-learning was used in combination with patient imaging (to provide structural information of the atria) and image-based modelling (to provide functional information) to design patient-specific catheter ablation strategies to guide clinicians and improve treatment success rates. The Q-learning algorithm was applied to an ablating agent that moved around a 2D late-gadolinium enhancement magnetic resonance imaging-based left-atrial model, applying catheter ablation to create non-conductive lesions that terminate AF, and learning through feedback imposed by a reward policy. The models included main structural features of the left atrium, such as pulmonary veins, and employed advanced image-processing techniques to represent patient-specific fibrosis distributions and computational modelling to simulate AF scenarios. The agent achieved 84% success rate in terminating AF during training and 72% success rate in testing. The AF recurrence rate after attempts to re-initiate AF in the two-dimensional atrial models after catheter ablation was 11%, suggesting the potential for large improvements on the existing approaches [107].

##### Treating hypertension in patients with type 2 diabetes mellitus

Q-learning was used to develop a RL system for dynamically recommending treatment for hypertension using EHR data of 14,934 hypertensive patients with type 2 diabetes mellitus from the Korean National Health Insurance Sharing Service (NHISS) between 2003 and 2013 [108]. The reward function was based on quality-adjusted life years (QALYs) and complications, body mass index, age, the period of hypertension, blood pressure, and the cost of medications which each impacted upon QALYs. The state of the patient was represented by five different categorised factors, including the presence of hypertensive-related complications (yes or no), age (less than 55 or 55 + years old), time since diabetes onset (0, 5–8, 9 + years), body mass index (< 18, 18–25, 26 + Kg/m^2^) and blood pressure (pre-hypertension, stage 1, or stage 2 hypertension). Each of these were categorised to create 2 × 2×3 × 3x3 = 108 different possible patient states. There were 14 possible medication actions to choose from consisting of either mono, dual, or triple therapies. The overall concordance of the models recommended actions with the doctor’s prescriptions was 85.2 and 81.5% for male and female patients, respectively. Blood pressure and the rate of hypertension related complications including heart disease and chronic kidney disease were inversely related to the concordance rates, suggesting that currently prescribed treatments that were in line with the model’s recommendations resulted in fewer adverse outcomes.

##### Treating patients with type 2 diabetes mellitus and hypertension

Double DQN models, each consisting of separate target and evaluation networks and using experience replay was applied to a dataset of 152,527 diabetes patients containing 415,707 records to recommend anti-glycaemic, anti-hypertensive, and lipid-lowering treatments. Separate DQN models were developed for hypertensive and lipid-modifying agents. For diabetes, there were 13 possible actions including adding an oral antidiabetic medication, increasing, or decreasing dose, and changes to insulin treatment. The reward function for the glycaemic DQN was based on the levels of glycated haemoglobin being < 7% after 3–6 months of treatment, the occurrence of hypoglycaemic events, and complications or death. A total of 178,489 visits were included to evaluate the short-term clinical outcomes of glycated haemoglobin control. Of these, 78,670 patient visits (44.08%) recorded treatments that were concordant with the DQN recommendations, and 99,819 (55.92%) were non-concordant, and medication patterns were more likely to be non-concordant for patients in worse health. Causal regression analysis to estimate the effects of medication concordance showed a 73%, 26%, and 28% higher odds of experiencing glycated haemoglobin levels < 7%, blood pressure < 140/90 mmHg, and LDL-cholesterol < 2.6 mmol/L, respectively, for patients receiving treatment in accordance with the model. There was also an inverse relationship between prevalence of adverse events and concordance between patient treatment and model recommendations.

##### Precision medicine in breast cancer

A novel personalized online-policy ranking system called Proximal Policy Optimization Ranking (PPOR) was developed using an off-policy actor–critic network to rank the suitability of different cancer drugs for individual cell lines. The focus was on determining the best treatment amongst a range of available treatments rather than simply predicting drug response for separate drugs and cell lines (patients). An actor–critic framework allowed the “actor” to be influenced by the evaluation signal from the critic network (clinicians’ actions) to ensure safe actions, in addition to also allowing continual improvement using sequential learning with continually updated data [87]. A LSTM RNN was included in the framework to capture the longitudinal dependence of the temporal records of patients. A metric called the normally discounted cumulative gain (NDCG) was used for evaluation, acting as a long-term reward function that captured the long-term effects of the ranking policy. The output for the model was the probability of selecting given treatments for the current state which depended on the cell line and the ranking position of the current treatment. Models were evaluated in a breast cancer cohort from The Cancer Genome Atlas Program and outperformed both state-of-the-art SL and recommendation algorithms and recommended treatment options that were in-line with results from recent cell-line specific randomised controlled trials.

##### Hierarchical reinforcement learning for prostate cancer treatment

Radiation therapy treatment plans involve many treatment planning parameters including weighting factors, dose limits, and volume constraints defined for treatment targets and organs at risk, and the values of these parameters critically affect the resulting plan quality. Treatment plan quality often depends on experience of the planner and available time and hence, fully automated approaches are strongly desired. Using prostate cancer intensity modulated radiation therapy (IMRT) and stereotactic body (SBRT) treatment planning as the testbeds, a hierarchical intelligent automatic treatment planning (IATP) framework was developed to improve treatment plan scores compared to previous frameworks [109]. The hierarchical virtual treatment planner network (HieVTPN) consisted of three separate RL networks that were responsible for making decisions at the structure, planning and adjustment action levels. Using a dataset of 74 training patient cases with 10 used for training, 5 for validation and the remaining 59 for testing, 500 random treatment plans were generated per patient, and each was fed to the HieVTPN for adjustment of the treatment plan parameters. The HieVTPNs for prostate IMRT and SBRT were able to generate high-quality plans, achieving average plan scores of 8.76 out of 9 and 137.89 out of 150, respectively. The decision-making behaviours of the HieVTPN were also understandable and generally agreed with human experience.

##### Causal dynamic treatment regimes for cancer

Dynamic treatment regimes (DTRs) consist of a sequence of decision rules to determine treatment assignment to patients based on the evolving treatments and covariates’ history. DTRs are particularly effective for managing chronic disorders such as cancer, diabetes, and mental illness, and are a key aspect of personalized decision-making. The development of a causal RL agent was motivated by the need to develop more efficient RL models which may at times not converge to the optimal policy with abundant observational data that may assist the heuristic process. By combining an online policy-based RL algorithm with observational albeit confounded data, informative causal bounds were determined that enabled a more efficient RL algorithm to be developed which learned the optimal DTR. The causal RL was developed and tested using randomly generated data for a DTR for alcohol dependence and data from a two-stage clinical trial for cancer. The causal RL model consistently outperformed algorithms that did not use causal bounds based on the cumulative regret function which measures how frequently the agent does not choose the optimal policy [110]. The study is one example of how causal RL models can improve on non-causal RL models by due to more efficient learning.

## Probabilistic algorithms

### Association rule mining algorithms

Algorithms for association rule mining aim to find those combinations of different items that occur more frequently than others such as finding hidden patterns in medical text data to help predict future events. Association rules are of the form IF *X*, THEN *Y* (*X* → *Y*), where *X* is termed as the antecedent of the rule and *Y* is the consequent. In a dataset of patient notes, item *X* might be diabetes and item *Y* might be hypertension. The dataset is searched to find frequent “if–then” patterns (for example if diabetes is present, then hypertension is also present) and metrics including support, confidence, and lift are used to define the degree of uncertainty in the rules. Support indicates how frequently the combination of a pair of items appears in the dataset, and confidence is the conditional probability that a third item will be present given that a given item-pair is present. Lift or lift ratio defines the ratio of the probability that the items occur together to the probability of them occurring independently.

#### Examples of association rules in medicine

Medication-disease patterns, symptom–disease patterns, and disease–disease patterns were extracted from 309 medical transcription files [111]. Natural language processing (NLP) was used to extract individual words which were matched with ontology databases to segregate medical terms. Multi-criteria decision analysis was first used to find the optimal set of association-rule algorithms, with the strongest rules generated from these then filtered according to confidence and lift. The top rules for medicine–disease, disease–disease and symptom–disease extracted were the use of the angiotensin converting enzyme inhibitor Accupril (quinapril)-hypertension, diabetes-hypertension, and anaemia-diabetes, respectively. The study demonstrated the validity of the approach given known clinical associations and the ability to predict disease development by considering disease–disease and symptom–disease rules.

### Probabilistic matching algorithms

Randomized algorithms employ a degree of randomness as part of their logic or procedure. In medicine, probabilistic matching is the preferred method for matching large data sets or when many attributes are involved in the matching process and determine the overall likelihood that two records from different databases are matched to the same individual. The success of linking EHRs depends on the number and quality of fields available for linkage and the linkage method used. Probabilistic algorithms may also be more suitable for EHRs than deterministic algorithms since EHRs have data entry errors and can therefore obtain a higher proportion of linkages.

#### Examples of probabilistic algorithms in healthcare

##### Probabilistic versus deterministic matching for electronic health records

A lack of information from randomized controlled trials on the safety and the efficacy of therapeutic interventions has promoted the use of EHRs in the U.S. for obtaining reliable evidence in large and representative populations including that of interventions during pregnancy. However, few health care systems in the U.S. have reliable mother-infant linkages available, with successful linkages obtained for only 74% to 99% of deliveries [112]. Deterministic algorithms typically require exact of near-exact matches on pre-specified identifiers. The accuracy of probabilistic algorithms and deterministic algorithms in linking mothers and infants were compared using surname, address, and dates of birth and delivery for 14,449 deliveries including 14,866 children amongst women enrolled in the Group Health Cooperative healthcare system in Washington State, USA [112]. The probabilistic algorithm improved the proportion of successful linkages and accuracy compared to the deterministic approach, with 84.1 vs. 74.5% sensitivity and 99.3 vs. 95.7% positive predictive value.

##### Probabilistic matching versus referential matching for electronic health records

Referential algorithms require demographic data from multiple sources [113]. Referential matching, an increasingly popular approach, instead uses large collections of demographic records, including information on past addresses, common names, and other demographic data such as phone numbers that change over time. In a comparison of the two approaches, Referential algorithms achieved a higher sensitivity and F-scores than probabilistic algorithms [113]. The use of referential sources enabled more complete capture of changes in combinations of demographics over time including name and phone number.

### Probabilistic graphical models

A probabilistic graphical model (PGM) is a way of representing a probabilistic model with a graph structure. The nodes in the graph represent random variables and the edges that connect the nodes represent the relationships between the random variables. Two of the most used PGMs are the Hidden Markov Model (HMM) and the Bayesian Belief Network or “Bayesian Network” (BN). The edges of a HMM are undirected, whilst in the edges of the BN graphs are directed, and the structure is generally referred to as a Directed Acyclic Graph (DAG). Both the probability distributions for the random variables (nodes) and the relationships between the random variables (edges) are specified subjectively, with the model then capturing the “belief” about a complex domain. Node dependencies are captured via the directed edges, with missing connections defining conditional independencies. BNs can be generated using either expert knowledge or by being learned from data and then used to estimate the probabilities for subsequent events. They have previously been applied in biomedicine and healthcare, for decision support in diagnosis, prognosis, treatment selection and discovering functional interactions [114]. Recently, BNs have also been used to understand patterns of multimorbidity [115, 116], their trajectories [115], risk factors [117], and predicted outcomes [118].

#### Examples of Bayesian networks to model multimorbidity

##### Modelling comorbidity trajectories

An UL approach that relied on neither medical experts nor medical literature, was used to construct BNs to mine for patterns of comorbidity evolution in a population of ~ 250,000 patients within the Department of Veteran Affairs in the U.S. [115]. The model performed similarly to a supervised and semi-supervised models that used medical expert knowledge or sufficient literature. A longest path algorithm (LPA) was used to mine major trajectories of comorbidities and identified that the most probable sequence of comorbidities between the emergence of substance abuse in year 1, and substance abuse in year 5 contained recurrent substance abuse across different years. This trajectory included 10% of all patients and suggested that a history of substance abuse is the major predictor of future substance abuse problems. The method also enabled identifying individuals at greatest risk for adverse outcomes including suicide and early mortality.

##### Identifying factors associated with multimorbidity

BNs were used to rigorously define and model varied aspects of multimorbidity to develop computerized decision support systems for personalized prescribing and to provide a framework for modelling interactions between multiple diseases [116]. The same authors used structure learning in BN to identify the role of critical risk factors including age, gender, smoking, and alcohol abuse in the pathogenesis of co-occurring malignant tumours. The study also demonstrated that structure learning in BN can be used for identifying critical factors of associated comorbid diseases where the role of such risk factors is less obvious [117].

##### Predicting lifestyle activities and health events

A BN was used to machine-learn a model for predicting vital (blood pressure, body mass index and cholesterol) and lifestyle parameters (medication usage and activity level) using data extracted from TILDA, The Irish Longitudinal health study of the older Irish population [118] which includes 8,504 individuals aged ≥ 50 years. The BN model represented patients with multimorbidity through several interconnected dimensions, i.e., demographics, medical factors, self-reports, and behavioural factors. To machine-learn the BN structure, the A* search algorithm was used to search for a shortest path [119]. In this demonstration project, the tool predicted vitals and goals (medication usage and activity level) with high accuracy even in the presence of missing data.

## Graph machine learning

Graphs are a common method for representing complex relational data in systems such as social media, biological protein networks, transportation, and healthcare systems. There is a growing interest in graph machine learning (GML) which involves the application of ML to graphs to extract knowledge from the graph and to also make predictions [120]. GML, also known as graph representation learning, is used widely in many industries including analysing the supply chain, fraud detection, providing recommendations, and drug discovery. The objective of GML is to obtain vector representations of the graph entities (e.g., nodes, edges, and subgraphs) to facilitate downstream tasks including community detection, node classification and link prediction. GML can play an important role in the performance of these downstream tasks [120].

A graph consists of a series of nodes and edges linked together to form relationships. The nodes might be a single type of entity such as patients, or they might include different entities such as patients and symptoms or patients and diseases (bipartite graphs). The edges that link the nodes demonstrate that a connection exists, and the lack of edges demonstrate the lack of a relationship. This set of relationships can also be represented in matrix form as a set of 1 and 0 s. Often and especially in a very large network, the proportion of zeros increase since most nodes are not connected. This happens because as the graphs grow, the average degree centrality (the number of connections that a node has) grows much slower or not at all. This is the case for social networks where a limit on the number of meaningful relationships exists and in graphs of user purchases for recommendation systems. To be able to use such sparse structures for ML requires that the matrices are compressed in some way. It is this compression that is the fundamental concept of GML which aims to capture the relationships in the network into an embedded graph and to also use the learned features from this embedding for future prediction using separate algorithms or the same algorithm.

**Fig. 6:**
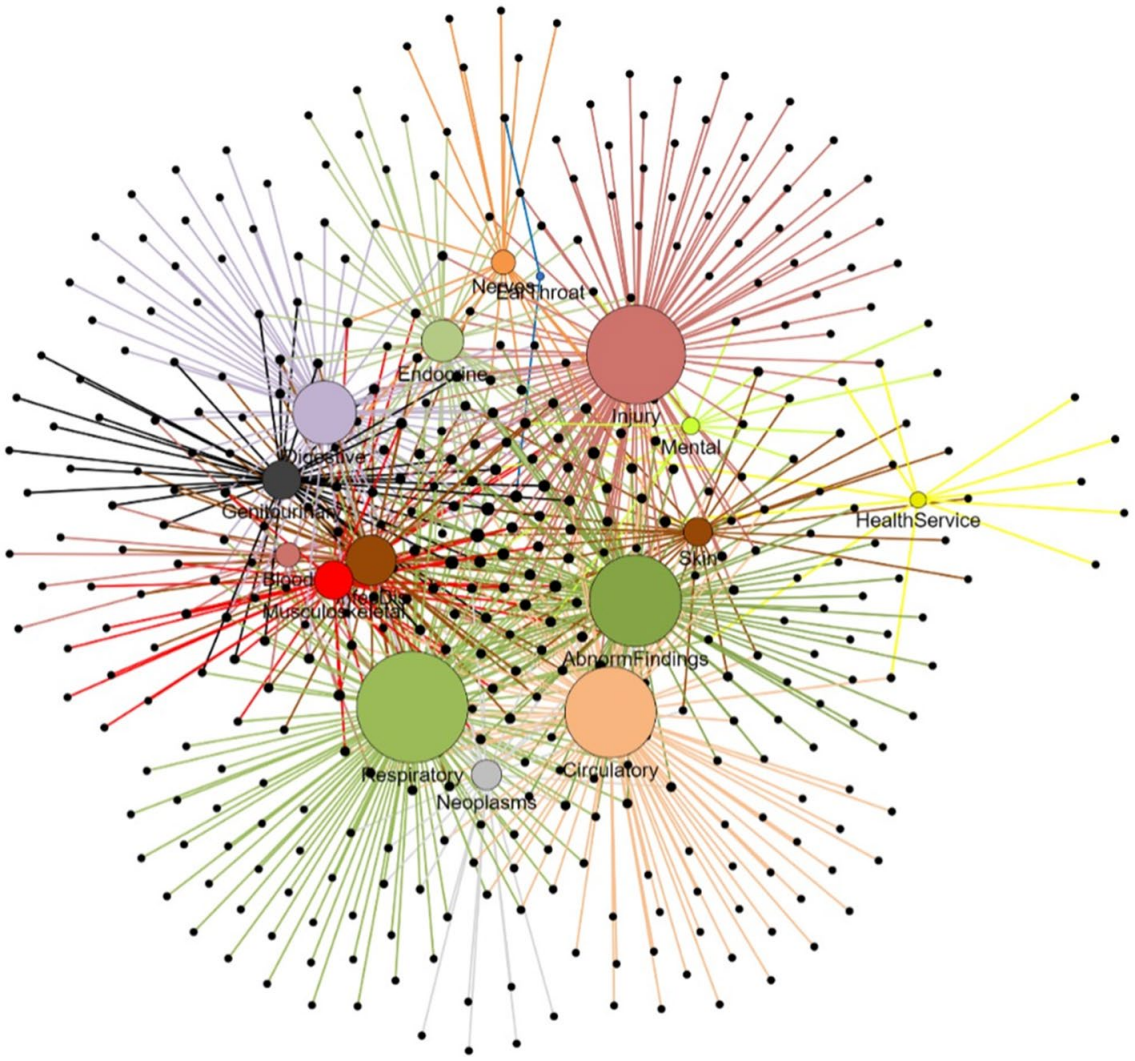
A graph network of patients and their conditions (ICD-10 Disease Chapters). Patients that are closer to one another are more similar. For example, patients in the centre of the network share more common diseases. Similarly, ICD-10 Disease Chapters that are closer together can also be thought of as more similar since they tend to co-occur in patients more often

An example of a graph with different node types (Patients and ICD-10 disease chapters) is shown in Fig. 6. Edges in the graph represent the medical diagnoses of individual patients (black dots). Patients in the centre of the network have more comorbidities than patients at the edge of the network, and their closeness also suggests a greater similarity. Similarly, disease chapters that are closer together are more likely to be found together in the same patient and it is likely that they may share common aetiologies and disease pathways. This and similar information can be extracted from the graph using graph modelling which takes various forms and levels of complexity ranging from extracting basic information on node and edge properties using graph algorithms to building fully end-to-end Graph models that can be used for future node and edge prediction.

### Graph algorithms

Graph algorithms are good first choices for understanding the properties of the graph with hand-crafted new features such as degree-centrality and betweenness centrality which are then used to train ML classifiers for disease prediction. Algorithms include PageRank (to rank nodes for their centrality), Louvain for graph-community detection, Dijkstra's shortest path for finding the shortest path between nodes and similarity algorithms to compute the similarity of pairs of nodes based on their neighbourhoods or the properties of the nodes such as patient features. For example, network-specific features including degree centrality, eigenvector centrality, closeness centrality, betweenness centrality, and the clustering coefficient, were generated from a patient network for the prediction of incident diabetes using random forests, NNs and other SL algorithms in 1,028 patients with diabetes and 1,028 patients without diabetes [121]. The newly generated features were more predictive for incident diabetes than demographic and clinical features. Similarly, patient–patient network and disease–disease network features accounted for 25.7% and 27.3% of feature importance in predicting hospital length of stay, compared with 15.3% and 31.6% for baseline (e.g., age and gender) and historical features (e.g., length of stay for previous admissions) [122].

### Graph embeddings

Graph embeddings are a form of representation learning in which numeric or binary feature vectors are generated to represent the nodes, relationships, paths, or entire graphs in a network. This provides a low-dimensional feature space and an effective solution for graph-related downstream tasks. The basic idea of node embedding is to generate a vector for each node such that the similarity between vectors approximates the similarity between the original nodes in the graph (geometrically and/or feature-wise). The different embedding techniques can be described as NN or non-NN based. The latter include graph kernels, matrix factorization, shallow models, and non-Euclidean models [120]. Established algorithms include Node2vec (random walk based), FastRP (using random projection and matrix operations), and HashGNN (hashing function architecture). The embedding process involves the adjacency matrix of the N graph nodes (an N x N matrix) being compressed into two-dimensional embedding vectors (an N × 2 matrix) in such a way as to ensure that the two-dimensional vectors representing the nodes cluster together in two-dimensional space and still reflect the graph community structure. Larger real-world networks with millions or even billions of nodes will typically have more than two dimensions (128 to 256 or higher) to represent larger real-world graphs. Compression of the matrix into the embedding vectors is performed such that the important signals in the graph structure are still maintained in the embeddings for downstream tasks.

### Graph neural networks

Graph neural networks (GNNs) are fully end-to-end graph embedding and prediction models using DL methods on graph-based data [123]. They have been used for assistance with clinical diagnosis of diabetes [124], disease prediction [125], disease prediction in patients in the intensive care unit (MIMIC-III dataset) [126], and a disease-diagnosis service for online users [127]. GNNs start by transforming the graph data into intermediate embeddings, which are provided to a final layer aligned to a prediction task including supervised learning (node property prediction, link prediction, graph property prediction), and unsupervised learning (clustering, similarity). Alternatively, they can be used to output embeddings that represent new features, and which infer the most important information from the graph. By training a model in addition to generating embeddings, GNNs are inductive and provide predictions on new data. Their weaknesses include high complexity, scaling difficulties, and low interpretability and explainability. In an overview of Graph ML techniques for disease prediction, GNN-based models were found to have superior performance compared to traditional ML techniques [123].

### Knowledge graphs

The term knowledge graph (KG) was first coined by Google in 2012 when they developed for use in their next-generation search engines which recognise not only the objects in a search but also the relationship between them. Although there exists no formal definition of KGs, they are generally understood to be “a graph of data intended to accumulate and convey knowledge of the real world, whose nodes represent entities of interest and whose edges represent relations between these entities” [128]. Whilst widely adopted for use in NLP tasks [128], KGs are increasingly used to represent different relationships such as those existing between diseases and medicines. By integrating patient records into multiple graph networks of proteins, diseases, drug compounds, molecular, and genes, KGs can be used to generate predictions tailored to individual patients. Patients with similar comorbidity phenotypes likely share disease mechanisms with other patients if for example their nodes have common gene and protein neighbours as other patients in the same disease cluster [129]. Incorporating biological knowledge in health datasets is likely to have broad applicability to many common diseases in the older patient population.

#### Examples of knowledge graphs

Using medical knowledge data gained from 29 publicly available sources, a heterogeneous network (Hetnet) KG of 11 node types (including compounds, genes, proteins, diseases and symptoms) and 24 relationship types (including compound–disease, compound–gene, gene–disease) was created to calculate the probability of each of 1538 compounds being a candidate treatment amongst 136 diseases and a total of 209,168 different compound-disease pairs [130]. The complete KG consisted of 47,031 nodes and 2,250,197 relationships. Amongst the 136 diseases there were 755 known (existing) treatments and 29,044 non-treatments (compounds not currently used to treat a disease). The degree-weighted path count (DWPC) was used to estimate the prevalence of compound-disease metapaths (of length 2 to 4), and 123 of these were used as features together with node-degrees for 14 meta-edges in training a regularised logistic regression model to calculate the probability of a compound being a treatment. Positive ΔAUROC's for metapaths indicated the paths that occurred more frequently between treatments than non-treatments and which are indicative of drug efficacy. There were 1206 metapaths of length 2–4 considered in the model, and of these 709 were significant for ΔAUC. Amongst them, 259 included a compound-binds-gene metaedge. An overall AUROC of 97.4% demonstrated high performance in detecting known treatments and the same model performed well in validation datasets (85.5 and 70.0%). Examples for epilepsy and nicotine dependence verified the high ranking of exiting treatments and clearly showed the properties that made other non-treatments likely candidates for drug repurposing.

The biomedical KG known as SPOKE (Scalable Precision Medicine Open Knowledge Engine) currently connects information from 41 biomedical databases and contains more than 27 million nodes of 21 types and more than 53 million edges of 55 types [131]. SPOKE is implemented as a Neo4j Community instance and built weekly from scratch by a series of custom Python scripts that download from each resource. In a study designed to assist in early detection of multiple sclerosis, SPOKE was embedded into EHRs of more than two million patients to uncover the hidden patterns of information existing within biomedical knowledge and patient records [132]. A modified version of the PageRank algorithm was used to embed the EHRs into SPOKE resulting in high-dimensional knowledge-guided patient health signatures called Propagated SPOKE Entry Vectors (PSEVs) of the graph nodes. Each PSEV represented a different node type (disease, symptom, side effect, compound, pharmaceutical class, gene, protein, gene ontology pathway, and anatomy) and each PSEV contained the same number of data points as nodes in the graph (~ 400 K). The PSEVs were used as new features in a random forest algorithm that improved diagnosis of multiple sclerosis for 5752 patients at three years before diagnosis (AUC = 0.83 vs. 0.60 using only EHRs). The study also allowed insight into the biological drivers of MS.

The same SPOKE KG was used for the early detection of Parkinson's disease [133]. In a random forest classifier, AUC accuracies of 0.77, 0.74, and 0.72 were obtained at one, three, and five years before diagnosis respectively, and accuracies of 0.74, 0.70 and 0.66 in a validation cohort. These were all higher at each time-point than when only EHRs were used (0.67, 0.63 and 0.56 at1, 3, and 5 years, respectively).

#### Other examples of graph machine learning in healthcare

##### Diagnosis of diabetes based on electronic medical records

Deep GNNs were used to develop a multi-relational graph of patients which could learn to diagnose diabetes for use as a clinical decision support tool [124]. EHR data for 2000 patients from a major metropolitan centre hospital in north-western China and graph structure learning were combined to construct a patient multi-relational graph, which abstractly described the relation between different entities in the EHRs including patient demographics, diagnostic information, laboratory tests and medicines. An original subgraph, overall feature graph, and a higher order semantic graph were fused to generate a higher quality heterogeneous graph containing the complex interactive higher order semantic information of the patient multi-relational graph. The structure of the heterogeneous higher order graph was then used for training a GNN. The model improved the AUC for diabetes prediction from 76% using a standard GNN to 92%, demonstrating that division of the multi-relational graph into separate components could create a higher order semantic graph that incorporates complex interactions medical entities and improve disease prediction.

##### Knowledge graphs to identify new drug indications

A novel heterogeneous information network (HIN) graph representation learning (HINGRL) algorithm that considered both network topology and biological knowledge was developed to identify new indications for drugs using graph representation learning techniques [134]. The HIN integrated drug–disease, drug–protein, and protein–disease biological networks with the biological knowledge of drugs and diseases. Different representation strategies were applied to learn the features of the nodes in the HIN from the topological and biological perspectives. A random forest classifier was then used to predict unknown drug–disease associations based on these integrated drug and disease features. The HINGRL outperformed three other state-of-the art algorithms proposed for drug repositioning, with AUC of 0.8835 and 0.9363 for separate benchmark datasets with labelled data for known drug–disease associations.

##### Predicting adverse drug reactions using disease–drug networks

A GNN was developed to improve the prediction of post-marketing adverse drug reactions (ADRs) by learning node representations of a heterogeneous drug–disease graph from 12 years of healthcare claims data. Nodes represented the drug and disease medical codes and the relationships between them were generated using edge-weight data. The GNN aggregated information of each drug/disease node from the graph with the weighted sum of neighbouring node features in previous GNN layers being used as node features for subsequent layers. The performance of the algorithm for predicting drug-ADR pairs was superior to that of a logistic regression model and neural network (AUC = 0.795 versus 0.631 and 0.739, respectively). ADRs were learned with minimal data processing and using well-established medical terminologies without requiring case-by-case feature engineering and domain expertise. Combing several forms of the algorithm also predicted ADR's not present in the database showing its capacity to supplement the ADR database [135].

##### Predicting length-of-stay in patients in the intensive care unit

The relational information from disease diagnoses was used to predict patient length-of-stay by connecting similar patients in the graph. A hybrid model combined Long Short-Term Memory networks (LSTMs) for extracting temporal features and GNNs for extracting the patient neighbourhood information. The LSTM-GNNs outperformed the LSTM-only baseline model with the study highlighting the potential for exploiting information from neighbouring patient cases using graph neural networks and EHRs [136].

## Natural language processing

### Medical text data extraction

It is estimated that as much as 80% of the information in EMRs is locked within clinical notes from multiple EMR sources. This provides huge volumes of data and the foundation for improved healthcare to patients, but the manual review of such volumes is unrealistic in terms of both cost and time. Natural language processing (NLP) algorithms are used in recognizing key elements of spoken and written language and for extracting semantic meaning. Text has many advantages over structured data including the capturing of a patient's narrative, better describing clinical findings and to provide quantified diagnoses since disease codes do not easily accommodate diagnostic uncertainty [137]. The information contained in text data is therefore likely to provide enhanced information from structured EMR data alone.

NLP algorithms can be broadly classified into low-level pre-processing tasks such as the identification of keywords, sentence boundary detection, sentence tokenization, stemming (reducing words into a root form) and lemmatization (to map similar words to a token) [128], and higher-level tasks that include the extraction of topics and meaning from unstructured spoken or written input. Early approaches to NLP were rule-based linguistic approaches, using pre-defined rules followed in a computer program. Since 1980, ML approaches using UL and SSL were developed, and since 2010, representation learning, and DL have been widely employed. Especially within an industry as critical as medicine, it is crucial that algorithms incorporate domain-specific knowledge into the solutions, to ensure essential texts or words are not lost in translation. Reassuringly, the use of NLP on unstructured data has achieved similar levels of accuracy in predictive performance to that obtained using structured EMRs. Moreover, combining both structured data including laboratory reports [128] and unstructured EMR data has been shown to considerably improve predictive performance for case-detection [138].

#### Examples of medical text data extraction in geriatric medicine

##### Maintaining cognitive function in older adults

An inquiry-based cognitive training application using NLP and running on mobile devices was used to stimulate cognitive function in older adults. The app continuously acquired personalized, inquiry-based information aligned with users’ interests from the media followed by the generation of multiple-choice questions, their answers, and related distractors [139]. The app used generative modules composed of Microsoft’s Azure Text Analytics API to extract data from the media and to relate it to other media. A series of bidirectional encoder representations from transformers (BERT), convolutional neural network (CNN), and sequence-to-sequence NNs were also employed to generate multiple-choice questions, their answers, and related distractors of the media content as well as for summarizing that content.

##### Prediction of atrial fibrillation

The prediction of AF using EMRs has generally been poor. In this study, NLP was used to provide additional features for 5-year prediction of AF amongst 39,051 older primary care patients [140]. Narrative text data were available as health care provider progress notes, visit notes, history and physical notes, discharge summaries, laboratory notes, and cardiology test reports, which were processed to extract information using a published approach [141], and Narrative Information Linear Extraction, an NLP package for EHR analysis [142]. First, a dictionary of terms were mapped to concepts in the Unified Medical Language System, for example, the terms “atrial fibrillation” and “auricular fibrillation” were both mapped to a unique identifier (C0004238). Free text from clinical notes were processed using NLP to count the number of positive mentions of each concept unique identifier, while disregarding negative mentions, such as “no evidence of ….”. Patients with at least one positive concept unique identifier match at baseline were considered to have the feature. ICD-9 and ICD-10 codes and algorithmically detected identifiers were also used to detect a range of comorbidities. When the ICD codified EMRs were combined with the narrative data the C-statistic improved marginally from 0.729 to 0.735 in the validation cohort, and from 0.738 to 0.750 in external validation, demonstrating perhaps both the difficulty in predicting 5-year AF and the limited improvements in performance that adding NLP can be expected to make when comprehensive coded data is already available.

### Topic modelling

Topic models are a group of unsupervised NLP algorithms that calculate the probability of a word given a topic, and the probability of a topic given a document. Essentially, they are a feature reduction technique, mapping the original words and sentences of a document into underlying latent topics, allowing rapid inference of the underlying text. Many different topic models exist with the choice depending on the level of predictive performance and the level of interpretability required for the task. Typically, classification models are interpretable if they can indicate the weights that have been assigned to each input feature. Text classification models typically involve a representation step in which the text strings are represented numerically (known as embedding), and a classification step in which a label is applied based on the embedding. Originally, the bag-of-words (BOW) approach was used to represent each word with one-hot encoding, however, this approach is not scalable and does not capture syntactic information. These limitations were overcome with the use of neural embeddings such as Word2Vec and have been widely used since their introduction in 2013. Neural embeddings represent words as vectors in a high-dimensional space with semantically similar words being located close to one another, enabling significant improvements in text classification. Other neural embedding models include BERT, Doc2Vec, Glove, and ELMO [143].

**Fig. 7:**
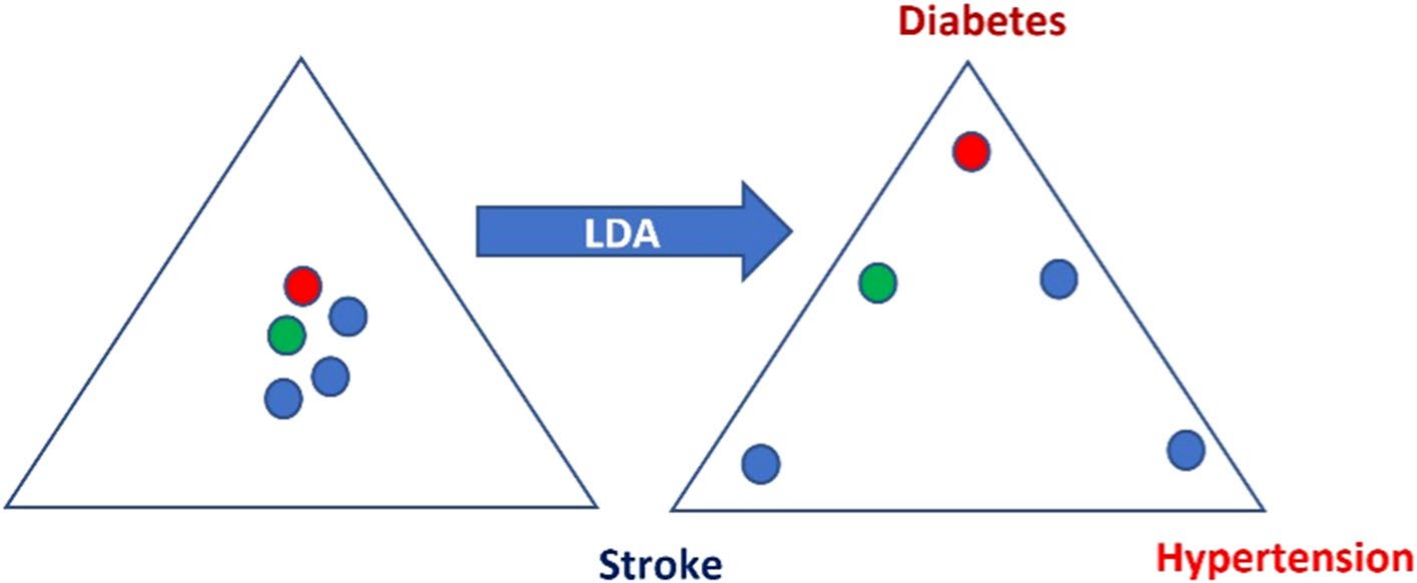
Example of Latent Dirichlet allocation (LDA) for a set of patient records. Each record can be classified as belonging to a latent disease grouping. Here, there is a high probability of the red record being a patient with diabetes. The classification probabilities for the red circle might be for example, 85% Diabetes, 7% Stroke and 8% hypertension. The green record would be classified as slightly more likely to be diabetes than stroke

Many topic models have their foundations in Dirichlet based models including latent Dirichlet allocation (LDA), latent semantic analysis (LSA), and probabilistic latent semantic analysis (PLSA) [144]. LDA recognises that documents typically contain more than one topic, and therefore provides a probability of a topic for each document such as diagnoses or medications prescribed during a patient’s visit (Fig. 7). LDA utilises a three-level hierarchical Bayesian structure (items, topics and documents) and was first used as a method for document modelling in 2003 [145]. LDA assumes that there exists a latent distribution of topics and that each topic can be seen as a probability distribution over the words. The same reasoning was used as the basis for other approximate inference algorithms including black-box inference methods. Autoencoding variational Bayes (AEVB) [146] trains an inference network, a NN that directly maps the BOW representation of a document onto a continuous latent representation. A decoder network then reconstructs the BOW from the latent document representation. ProdLDA and NeuralLDA were the first topic modelling algorithms to use AEVB inference methods. ProdLDA models a probability distribution by combining the output from several simpler distributions, whereas NeuralLDA uses a single mixture model. Other topic models focus more on dimensionality reduction than on considering the probability distribution of topics and words. Fuzzy latent semantic analysis (FLSA) is a dimensionality reduction technique using singular value decomposition (SVD) to project words into a lower dimensional space in a meaningful way. SVD transforms the original document-term matrix A into A = UΣV^T^ where U is a document-topic matrix, Σ is a matrix with a diagonal of singular values (weights), and V is the term-topic matrix. FLSA performs fuzzy c-means clustering on U to find different topics. FLSA-W is like FLSA but clusters on V rather than U, hence clusters on words rather than topics.

A comparison of 17 different topic models was performed to assess the ability to predict violence amongst psychiatric hospital patients using the clinical notes of 834,834 psychiatric patients presenting between 2012 and 2020 [143]. Encoding algorithms included ProdLDA which performed the best for predictive performance and performed well for interpretability. FLSA-W was the preferred model based on interpretability. Although LDA had high coherence scores, indicating similar words within a topic, many of the topics contained the same words making topic interpretability poor. LDA also performed relatively poorly for predictive performance.

#### Examples of topic modelling algorithms

##### Modelling of healthcare status for clinical decision support

A novel multiple-channel latent Dirichlet allocation (MCLDA) approach was used to model the latent healthcare status of patients using diagnoses, medications, and contextual information (age, sex and medical division) in healthcare data [147]. Using real-world data of ~ 1 million healthcare insurance claim records, MCLDA captured comorbidity structures, and linked them to the distribution of medications to identify pairings between diagnoses and medications based on the assigned latent groups. The resulting latent medication and disease groupings were more successful than other algorithms at predicting each other. Together, the results suggested MCLDA as being a strong candidate to use for detecting hidden medication and disease clusters in healthcare data, which can then be used for other prediction models.

##### Modelling the temporal trends of clinical care

LDA was used on longitudinal patient reports to learn latent topics and identify temporal patterns of clinical care in a cohort of brain cancer patients [148]. Patients with a minimum of 5 records were included resulting in a dataset containing 303 patients, 13,028 reports, 2,412,385 words, and 1,374 unique words. Labels for the identified topics included imaging diagnosis, surgical resection, radiation treatment and post-treatment imaging surveillance. Reports were pre-processed to remove stop words, rare and common words, and a set of medical stop words, such as “Dr.”, “report”, “dictated”, and “ID”. When reviewed by a neuroradiologist, the resulting topics and temporal patterns provided a valid sequence of clinical events. For example, the topic for radiation treatment was generally preceded by the topic for surgical resection of a tumour. The ability of the topic model to learn temporal-topic expression demonstrated the potential to self-learn patient groups and to predict the risk of events.

##### Developing precision medicine for diabetes

The use of topic modelling as a structure for clinical decision support for personalised diabetes management was explored by applying LDA to 1426 PubMed abstracts that contained information relating to diabetes-gene associations [144]. The Gensim Python package (2019) was used for the analysis. Using an automated topic coherence score to assess the similarity of words within a topic and to identify the ideal number of topics, four separate topics were identified amongst the keywords, and accounted for 33.9%, 26.6%, 26.4% and 13.1% of the corpus of words, respectively. Topic 1 was highly related to Maturity-Onset Diabetes of the Young, topic 2 was related to gestational diabetes, topic 3 was related to genetic mutations that affect diabetes, and topic 4 was related to pancreatic function. Personalised treatment recommendations could be achieved by matching new patients to the most suitable treatment based on their genetic and phenotypic profile and the modelling of patient-treatment reactions.

### Graph-embedded topic modelling

Graph-embedded topic modelling (GETM) combines KGs with embedded topics from topic modelling to enrich the topics. Raw medication and diagnosis data were used as a bag-of-words input for topic inference, and knowledge graphs were created separately from the same data to provide pre-trained graph embeddings [149]. The output from these two models were then combined to provide knowledge-enriched and more informative topic distributions. The learned patient topic mixtures were more predictive than raw features in predicting chronic musculoskeletal pain phenotypes. Using GETM also revealed known or potentially new condition-medication relationships.

### Large language models

Large language models (LLMs) are trained on large amounts of data including open sources on the internet, such as openly available medical texts, research papers, health system websites, and openly available health information podcasts and videos. LLMs, also known as ChatBots, are designed to generate new text and now typically contain many hundreds of billions of parameters. They function by tokenising existing sentences and words into word components and encode and then decode these tokens using what are called Attention mechanisms to combine tokens effectively when creating their answers. Attention mechanisms are used for sequence-to-sequence problems and to start, relied on RNNs. However, these initially suffered from an inability to retain information on the first tokens used in a sequence when dealing with long sequences. This problem was overcome in 2017 with the development of an algorithm architecture known as a transformer which consists of extracting features for each word using a self-attention mechanism to figure out how important all the other words are with respect to the aforementioned word [150]. Transformers learn in every step to focus on the right element of the input to predict the next output element, hence the term attention, and perform parallel processing. They are now the default architecture for LLMs.

Chat Generative Pre-trained Transformer 4 (ChatGPT4), an example of a LLM using a transformer architecture was released to the public on the 30^th^ of January 2023, providing a novel method of interaction with AI for the public as well as for clinicians. Since it was trained with the aim of achieving general cognitive ability, the training data included medical information, making it capable of also being used for medical documentation and with differential diagnosis. ChatGPT and similar models may not reference their sources making it difficult to determine the clinical veracity of their answers especially when there is no single answer to the given prompt by the user [151]. Although answers are often stated convincingly, artificial responses can be generated when the answer is unknown (referred to as “hallucination”) and can be difficult to detect by non-medically trained individuals. When the accuracy of Chat-GPT for diagnosis was compared to clinicians and lay-individuals using 48 vignettes, ChatGTP achieved 88% accuracy compared to 96% for clinicians and 54% for lay individuals [152]. Although such results are promising, concerns regarding privacy (ChatGPT collects information on location and IP address) and the potential for the distribution of patient information in the cloud has led to some health authorities restricting its use [153].

#### HuatuoGPT for medicine

Whilst recognising the need for caution, it is likely that ChatBots will eventually provide both better medical decision making and more time for patient interactions, resulting in improved patient outcomes [154]. LLMs designed specifically for Medicine (LLM4Med) are now being developed including HuatuoGPT, named after Chinese physician Hua Tuo [155]. HuatuoGPT incorporates ChatGPT and real-world medical data to bridge the gap between ChatGPT responses and those given by a clinician and can be used by individuals seeking an initial diagnosis, as well as by clinicians. Distilled data from ChatGPT is used as a base and additional real-world medical data injects medical knowledge. The algorithm combines the data with a transformer, SL and RL from AI Feedback (RLAIF), and leverages the strengths and mitigate the weaknesses of each type of data. The LLM aims to perform medical diagnoses, prescribe medications, provide medical and health advice, triage, and interpret medical reports.

## Recommender systems

Recommender systems aim to predict relevant items to users by building a model from past behaviour, and have been applied in multiple domains including e-commerce, social media, and advertising as well as health [156]. Within healthcare, health recommender systems (HRSs) have the potential to predict items such as health messages that will be relevant for individuals [157]. In medicine, medical information may be recommended to health professionals working with a patient and their health record or to patients inspecting their own personal health record.

The five types of recommender systems include collaborative filtering, content-based filtering, knowledge-based, comparison, and hybrid systems. Within healthcare, hybrid systems are by far the most employed [156]. In the commercial field, content-based filtering consists of assessing the similarity of previous items chosen by a consumer, and then recommending new items that have similar characteristics, for example movies of a similar genre. In collaborative filtering, the records of all user behaviours are recorded, and a user/item dataset that clusters users with similar preferences is built. The two major classes of collaborative filtering techniques are (i) neighbourhood methods, which predict the user–item association based on pre-defined user–user and item–item similarities, and (ii) latent factor models, which use matrix factorization to embed high-dimensional datasets into a low-dimensional latent space that captures user-item associations. In content-based filtering, pre-trained computer-vision or NLP models are commonly used for pattern recognition to gather as much information as possible on possibly only a few items previously used by a user.

The most modern recommendation systems use a hybrid of content and collaborative filtering that enabling the finer interactions between items and users to be learned. Hybrid Deep Learning algorithms are non-linear and can represent complex tastes over a range of items, including cross-domain datasets covering music, movies, and TV shows. The graph network of users and items are modelled using embeddings that are learned using both the collaborative filtering approach, and the content-based features.

### Recommender model examples in healthcare

In healthcare, examples of recommender models include recommending food items to diabetic patients [158], decision support for therapy [159], mental health apps [160], health-behaviour change [161], clinical order entry [161], treating COVID-19 [162] and cancer treatment [163]. In precision medicine, recommender systems can be used to predict the preferred treatment for a disease based on multiple patient measurements [164]. Personalised health recommender systems for recommendations on either lifestyle-related activities, nutrition, general health information and specific health conditions has recently been reviewed [165].

## Clinical implications and challenges

Our review has summarised the major ML domains and techniques being used in health research, with a focus on applications in geriatric care, highlighting the tremendous potential for improved patient health, clinical efficiency, and workflow. In particular, clinical decision making is often a highly complex process involving consideration of multiple interacting factors including contraindications, risk of adverse effects, and the likely response to treatment, restrictions on drug availability, clinical context, patient preference, provider bias, prior training, local medical practice disease and risk-factor interactions, and drug–drug and drug–disease interactions [8]. The hope and promise of precision medicine is to provide models that incorporate all this complexity whilst also tailoring treatment to the individual or patient phenotype [166]. This challenge can only realistically be achieved with data-driven complex algorithms [19], and requires both clinicians and patients to be accepting of transferred responsibility for diagnostic and treatment decisions from human to machine [167]. In addition to this fundamental mind-shift, many other more practical barriers must also be addressed to ensure that the potential benefits of AI in healthcare are not unintentionally offset by overlooking factors that might exacerbate rather than reduce disparities in healthcare due to social inequality [168]. Two areas of special concern are the use of potentially biased training data, and the need for better algorithm transparency.

### Bias in algorithms

Building more complex models using data-driven approaches on datasets with a high-dimensional feature space requires datasets that are sufficiently large and diverse to ensure that the algorithms are not trained with any blind-spots in the data. Without this fulfilment, the algorithms performance will not generalise well to populations, leaving accurate prediction for new subjects that match these clinical characteristics either difficult or impossible [169]. Blind-spots reflect what is often termed the curse of dimensionality, with the number of possible “blind-spots” increasing exponentially as the number of relevant (predictive) features with sparse data increases [169]. Causes of sparse data include the use of atypical data which is not physically possible, random sampling, and bias in the data collection which fails to include the relevant sample space. Assuming that the sparse data region is supported physiologically, algorithm performance may change dramatically, depending on the nature of the complexity of the underlying associations (linear or non-linear).

A high-profile example of non-representative data is the case of IBM’s Watson for Oncology system which was trained on high-dimensional historical patient data to make treatment recommendations for eight different cancer types. Watson was trained on small datasets consisting of between 106 ovarian cancers and 635 lung cancers and relied on data and treatment recommendations came from a single centre [8]. When used elsewhere, oncologists frequently reported lower concordance rates between their own and Watson’s recommendations. Although the recommendations that Watson learned from oncologists working at the Memorial Sloane Kettering Cancer Centre (MSKCC) might generally aid the affluent New Yorkers served by this hospital, they may be inappropriate for patients with very different clinical complexities. Rather than being only an isolated case, the problem of using large but non-representative datasets is widespread. In a review of 75 studies that trained DL models on image-based data using U.S. hospital patients [170], only 16 of the 50 U.S. States (32%) were included, whilst 68% of studies used data that included either California, Massachusetts, or New York. These states may have economic, educational, social, behavioural, ethnic, and cultural features that are not representative of other areas of the country, making it highly feasible that the predictions and treatment recommendations of the algorithms will be less accurate and suitable in these areas.

Whilst the potential for biased models from using non-representative datasets has long been known, the problem is dramatically magnified when using data-driven ML algorithms which also have complexity magnitudes higher than those of previous regression models. This is the reason that researchers, industry, and regulatory bodies must therefore now doubly ensure that training data mirrors the populations in which the algorithms will eventually be used. Researchers and data scientists should also carefully consider whether the available sample size can support the complexity of the proposed application, being aware of the negative log-linear relationship between sample size and accuracy [169, 171], meaning that whilst sample size requirements increase linearly with additional features when underlying associations are linear, they increase exponentially when non-linear. To mitigate these problems, model complexity should be limited during development, features should be robust and non-sparse, and the training sample should be unbiased. Finally, model deployment should be carefully monitored to detect potential drops in performance resulting from mismatches between the training and deployment data. A recent example where the deployed model performance fell far short of its training performance is the EPIC Sepsis proprietary Prediction Model (ESPM) used for sepsis identification in intensive care units employing the EPIC EHR system which is the leading EHR system in the U.S. [172]. A penalised logistic regression algorithm was used with EMR data from only three health systems in the U.S. Although the reported AUC during development was a reasonable 0.73 for sepsis prediction [173], a much lower AUC of only 0.63 was achieved in an external validation cohort [174]. In addition, amongst patients with an ESPM score of 6 or more which is in the recommended range for alerting clinicians, the number needed-to-treat to identify one additional sepsis patient was 8, high enough to cause alert fatigue, inappropriate triage, and unnecessary diagnostic testing, and antibiotic prescribing. This case is particularly relevant given that the model is being used widely as a clinical decision support tool for the early identification of sepsis in the intensive care unit. This example highlights the importance of providing greater details of model development and performance and for allowing open source access to data and code to allow independent validation [172]. The case also demonstrates how AI products can be embedded into healthcare systems, sometimes quite broadly, without the need for regulatory (e.g., Food and Drug Administration, FDA) approval of software, despite being within the bounds regulated medical devices, either as Software as Medical Device (SaMD) or as Software in a Medical Device (SiMD) [175].

### Algorithm transparency

Improved performance from ML is often achieved at the expense of increased model complexity, resulting in uncertainty regarding both the way they operate mathematically and in their predictions and decisions [5]. This so-called “black box” nature of complex algorithms which is often the trade-off for enhanced performance is often identified as the key for a lack of trust by clinicians and creates barriers for the adoption and implementation of AI into healthcare [176, 177]. DL techniques in particular are especially known for having limited transparency [178], which is a direct result of the non-linear structure and transformation of the original data during error back-propagation, an inherent feature of DL algorithms [19]. A Recurrent Neural Network (RNN) with a long short-term memory (LSTM) used to provide optimal treatment actions for patients with sepsis [92] can considerably enhance performance compared to regression and non-DL models but it cannot explain the reason for its treatment recommendations and clinicians must, therefore, learn to simply trust the algorithms when facing a potentially life-saving decision. Fortunately, many advances have been made in attempts to help better explain and also visualise both DL [179] and non-DL models. Encouragingly, the information on predictions generated from modern explainable ML methods is compatible with the traditional domain knowledge for disease prediction amongst clinicians [180].

Two popular models developed to explain the results of non-DL tree-based algorithms are feature importance plots and SHAPley values, which together provide transparency at both the overall and individual patient level [10]. Feature importance is the relative importance of each feature in generating the model's predictions and is calculated based on the overall reduction in the loss-function used during algorithm training. Feature importance for the various features is often visualised using a bar plot. SHAPley values are a weighted sum of the marginal contributions for each feature in the model and can provide both the overall mean effect of a feature, and the effect of each feature for a specific individual, which will vary according to the values of their other features. These methods for improved transparency are illustrated in a study examining the predictive factors for outdoor activity limitation (OAL) in the older community. Data from a cross-sectional survey of 6794 community dwelling adults aged 65 years and over with information on six different domain types (sociodemographic, health, physical capacity, neurological manifestation, daily living habits and abilities and environmental conditions) was used to develop an interpretable data-driven ML model to gain an understanding of the predictive value of multidimensional factors on OAL and to identify potential dimensions or factors for targeted interventions [181]. To provide improved transparency to practitioners, SHAPley relative importance bar plots were used to provide an overall ranking of the 20 most predictive factors, and individual SHAPley values for each subject were displayed in a bee-swarm plot to show the contribution of each feature to the predicted outcome for individual subjects. The XGBoost supervised classification model outperformed a logistic regression model (AUC = 0.918 versus 0.897) and the SHAP values demonstrated the ability to provide interpretation despite complex non-linear interactions in the XGBoost model.

In UL, the clustering of high-dimensional data also presents challenges when identification of the factors driving cluster membership is no obvious. Embedding algorithms including t-Stochastic Neighbourhood Embedding (t-SNE) can transform high-dimensional data into just two or three dimensions whilst at the same time preserving the similarity of the original data. Features of interest in the original sample space can be colour coded and applied to the t-SNE two- or three-dimensional scatterplot to see their influence in clustering. In patients with heart failure, t-SNE was used as a visualisation tool to show the association between higher chronotropic response and/or effort during a 6-min walk test and increased functional capacity [182].

## Regulation and adoption of clinical decision support tools

Under the current FDA regulation, CDS tools enable algorithms to be considered as either software as a medical device (SaMD), or as proprietary algorithms developed for clinical decision support. Whilst algorithms labelled SaMD require formal regulatory scrutiny by the FDA, proprietary algorithms developed within existing EHRs traditionally sit outside of the FDA scope [183]. Non-device CDS tools provide recommendations in clinical settings where the physician can review the basis for using the predictions. To clarify what this means, guidance provided in 2019 stated that the CDS must only “recommend” rather than “drive” decisions, and it is not intended for healthcare providers to rely primarily on such recommendations for making clinical diagnosis or treatment decisions [184]. Of course, the line between “drive” and “recommend” can be easily blurred leading to the development of CDS tools that do not require formal regulatory approval, but which can still be heavily relied upon during decision making [175]. When faced with a choice of using AI tools, clinicians will likely at some stage therefore need to use their own judgement rather than relying on regulatory approvals. In making the decision to both use and accept the model's recommendation, it should be remembered that all clinical decision necessarily comes with a certain level of uncertainty, sometimes an alarmingly high level of uncertainty that cannot be reduced to zero. Complex ML algorithms within the workplace can therefore be viewed, at least to some degree, as simply another unknown, albeit in a different form [8]. CDS tools are designed as tools to assist rather than override clinical judgement, and intuition should still be used to help identify obvious patient-state versus AI recommendation anomalies.

## Summary

This article has provided the reader with a comprehensive overview of the range of ML algorithms currently employed within healthcare research. Some of these algorithms are already employed within healthcare technology within aged care whilst others have not yet been deployed but have been applied within research settings and have demonstrated the potential for improvements in healthcare outcomes. Together, the various models encompass a phenomenal range of different applications, spanning a huge range of different medical areas, but all with the underlying aim of supporting clinicians and improving patient outcomes. Whilst the overall likely impact on healthcare is still seen by most as a hugely positive change, the new paradigm of AI in the healthcare industry has brought its own issues such as the potential for exacerbating inequities in healthcare and the potential for harm due to inadequate validation and lack of transparency. These and other problems require harmonic resolution with by the authorities that govern healthcare, the researchers and data scientists that develop the tools as well as the clinicians that use them.

Having explained the technical details of the numerous algorithms that abound in ML and how they are being employed in healthcare, this article has aimed to provide not only a picture of the present state of ML and AI in healthcare, but also the huge potential for further rapid advances given the constant development and refinement of each ML domain and their growing deployment into different areas. Combined with the largely untapped avalanche of new data available from EMR healthcare systems and that flowing from the internet of things medical devices, what we have achieved to date may yet be only a glimpse into how healthcare, particularly geriatric care, may change in the coming decades.
